# Characterization of Bud3 domains sufficient for bud neck targeting in *S. cerevisiae*

**DOI:** 10.1099/acmi.0.000341

**Published:** 2022-03-24

**Authors:** Madison N. Schrock, Yao Yan, Megan E. Goeckel, Erianna M. Basgall, Isabel C. Lewis, Katherine G. Leonard, Megan Halloran, Gregory C. Finnigan

**Affiliations:** ^1^​ Department of Biochemistry and Molecular Biophysics, Kansas State University, 141 Chalmers Hall, Manhattan, KS 66506 USA; ^‡^​ Present address: School of Biological Sciences, University of Utah, Salt Lake City, UT, 84112, USA; ^§^​ Present address: Department of Cell Biology and Physiology, Washington University in St. Louis, School of Medicine, St Louis, MO, 63110, USA; ^#^​ Present address: Department of Neurobiology, School of Medicine, University of Utah, Salt Lake City, UT, 84112, USA; ^¶^​ Present address: School of Medicine, University of Texas Medical Branch, Galveston, TX, 77555, USA; ^**^​ Present address: Memorial Sloan Kettering Cancer Center, New York, NY, 10065, USA; ^††^​ Present address: Department of Psychology, University of Kentucky, Lexington, KY, 40506, USA

**Keywords:** Bud3, septins, bud neck, fluorescence microscopy

## Abstract

The cytoskeleton serves a diverse set of functions in both multi- and unicellular organisms, including movement, transport, morphology, cell division and cell signalling. The septin family of cytoskeletal proteins are found within all fungi and metazoans and can generate three-dimensional scaffolds *in vivo* that promote membrane curvature, serve as physical barriers and coordinate cell cycle checkpoints. In budding yeast, the septins organize into polymerized filaments that decorate the division site between mother and daughter cells during mitosis; assembly of this structure at the ‘bud neck’ is critical for completion of cytokinesis and execution of numerous other cellular events. One such pathway includes bud site selection and the recruitment of proteins such as Bud4 and Bud3 that are responsible for promoting an axial budding pattern in haploid yeast. While Bud4 appears to be recruited to the septins independently of the presence of Bud3, it is likely that Bud3 can localize to the bud neck using both Bud4-dependent and Bud4-independent mechanisms. Furthermore, it remains unclear which precise domain or domains within Bud3 is/are both necessary and sufficient for optimal association at the septin structure. In this study, we examined the localization of GFP-Bud3 constructs in otherwise wild-type (WT) haploid yeast cells expressing Cdc10-mCherry using fluorescence microscopy; we tested a collection of N- and C-terminal truncations and fusions of separate Bud3 protein elements to identify the smallest domain(s) responsible for bud neck localization. We found that the coordinate action of the central amphipathic helix (residues 847–865) and a partially conserved C-terminal motif (residues 1172–1273) was sufficient to promote bud neck recruitment in the presence of endogenous Bud3. This domain is considerably smaller than the previously characterized C-terminal portion required to physically interact with Bud4 (1221–1636) and utilizes a similar mechanism of pairing membrane association, with a separate localization domain, similar to other non-septin proteins targeted to the division site during cell division.

## Introduction

The cytoskeleton within fungi and metazoans includes a component termed the septins that have a diverse set of roles at the cellular level [1, 2]. These proteins polymerize into longer filaments and superstructures that (i) influence membrane curvature, (ii) serve as a physical barrier between membrane-bound compartments and (iii) function as a three-dimensional scaffold on which scores of other non-septin proteins can bind and carry out information exchange [3–5]. In the well-studied budding yeast species *Saccharomyces cerevisiae*, there are seven total septin genes – five expressed during mitosis (*CDC10*, *CDC3*, *CDC12*, *CDC11* and *SHS1*) and two exclusively expressed during sporulation (*SPR28* and *SPR3*) [6, 7]. The essential mitotic septin subunits (with a ‘CDC’ designation) assemble into a core octameric structure consisting of a twofold axis of symmetry with the arrangement Cdc11-Cdc12-Cdc3-Cdc10-Cdc10-Cdc3-Cdc12-Cdc11 [8]. The non-essential Shs1 subunit can assemble into separate octamers and replaces the terminal Cdc11 protein to generate Shs1-Cdc12-Cdc3-Cdc10-Cdc10-Cdc3-Cdc12-Shs1 assemblies [9]. Septin octamers then bind laterally with other protomers and polymerize end on end to create long filaments and this assembly is critical for function; filaments can be arranged into numerous geometric shapes and structures, including arcs, gauzes, spirals, rings and hourglass shapes [10–12].

During the mitotic cell cycle, septins localize at the site of the emerging bud and decorate the ‘bud neck’ between mother and daughter cells; during cytokinesis, the septin collar splits into a double ring structure prior to the complete separation of the two cells [4]. Throughout this process, numerous non-septin proteins are recruited to the bud neck and septin superstructure. These include proteins from cell signalling pathways such as Hsl1, a kinase involved in the G2/M morphogenesis cell cycle checkpoint [13], Bni5, an adaptor protein linking the septins with the actomyosin contractile ring [14], components of the mitotic exit network such as Cdc5 [15], and a host of other protein modifying enzymes [3]. A diverse set of post-translational modifications of septin subunits or their associated binding partners is critical for regulation of septin assembly, physical geometry and information exchange among these cellular pathways [16].

One pathway that utilizes the septin structure at the bud neck is bud site selection; in haploid cells, an axial budding pattern occurs – choice of the next division site proximal to the previous site [17]. Previous studies have demonstrated that proteins Axl1, Axl2, Bud3 and Bud4 are responsible for determination of the bud site through activation of Cdc42 [18–22]. Genetic and biochemical data have demonstrated that Bud3 and Bud4 are recruited to and interact with the septin cytoskeleton [23–26]. Bud4 appears to have multiple domains sufficient for recruitment of the septin collar that may also act independently of Bud3 [24, 25]. The C-terminal regions of Bud3 and Bud4 appear to be responsible for their interaction [24, 27]. The complete mechanisms regulating Bud3 localization to the septin collar appear to be complex, and both Bud4-dependent and Bud4-independent targeting have been observed [27].

The precise domain(s) within Bud3 responsible for interaction with septin subunits and/or Bud4 (or other factors at the bud neck) remain unclear. A previous study suggested that three separate fragments (1–946, 674–1220 and 1221–1636) of Bud3 might independently provide targeting information to the bud neck [23]. However, when Bud3 fragments were expressed *in vivo*, they appeared to only weakly associate at the bud neck [23]. While it is possible that optimal Bud3 recruitment to the septin collar might require both Bud4-dependent *and* Bud4-independent modes, we suspect that, like other large multi-domain proteins at the bud neck, Bud3 may require the contribution of a membrane association domain. For example, our previous work demonstrated that Hsl1 requires both a central septin-binding domain as well as a C-terminal KA1 membrane-binding domain to assist in bud neck targeting [13]. Within Bud3, a central amphipathic helix (AH) is defined by residues 850–858, which is sufficient for membrane association [23]. Finally, limited work has been done on the C-terminal (1221–1636) domain of Bud3; yeast two-hybrid experiments indirectly suggested that a smaller region (1221–1466) may be sufficient for Bud4 interaction *in vivo* [25].

Therefore, in order to examine the molecular determinant(s) within the Bud3 protein responsible for bud neck targeting, we tested whether a collection of Bud3 fragments and fusion proteins were necessary and/or sufficient for its localization pattern. This included expression of N-terminally GFP-tagged Bud3 within yeast expressing Cdc10-mCherry and endogenous levels of both Bud3 and Bud4. A collection of N-terminal and C-terminal truncations were tested for co-localization with the septins using fluorescence microscopy and revealed that the N-terminus (residues 1–846) was dispensable for bud neck targeting. Additional constructs and fusions *in trans* demonstrated that coordinate action of the central AH domain (847-865) paired with a C-terminal fragment (1172–1273) was sufficient to promote localization to the septin collar. Finally, the contribution of the AH domain likely involved its ability to bind membranes, rather than septin association, as replacement with the C2 domain from bovine lactadherin was able to replace this region. Together, these results highlight a conserved motif found within the C-terminal Bud4-association domain and suggest that this smaller region of Bud3 (residues 1172–1273) is sufficient for targeting in the presence of both WT Bud3 and Bud4.

## Methods

### Yeast strains and plasmids

The *S. cerevisiae* strain and DNA plasmids used in this study are described in Table S1, Fig. S1 (available in the online version of this article). Creation of vectors expressing Bud3 fusions followed a general strategy utilizing *in vivo* plasmid assembly, lithium acetate transformation and selection [28]. Briefly, a parental vector was first constructed containing the promoter of *CDC11*, GFP coding sequence, a *SpeI* restriction site, the *ADH1* terminator and the hygromycin resistance cassette (Hyg^R^). Yeast were co-transformed with (i) the aforementioned plasmid linearized with *SpeI* enzyme and (ii) PCR-amplified fragment(s) of the *BUD3* gene containing flanking homology to both GFP (upstream) and the *ADH1* terminator (downstream) using a high-fidelity polymerase. Confirmation of proper assembly included diagnostic PCRs on clonal isolates and Sanger DNA sequencing (Genscript). For several vectors (Table S1), a similar strategy was used with a parental plasmid containing only the *CDC11* promoter; in this case, all additional components were introduced on separate PCR fragments such as GFP and the 3′ drug resistance cassette.

### Culture conditions

Two types of nutritional medium were used to grow yeast: rich YPD included 2 % peptone, 1 % yeast extract and 2 % dextrose, whereas minimal drop-out synthetic-based mixtures included a yeast nitrogen base, ammonium sulphate and amino acids. Solutions and sugars were filter-sterilized prior to use. Yeast cultures were grown with constant circular shaking in a temperature-controlled unit at 30 °C; agar plates were incubated at 30 °C.

### Fluorescence microscopy

Yeast cultures were grown overnight in synthetic liquid medium (SD-LEU) overnight at 30 °C, back-diluted into YPD, grown for an additional 4 h, harvested by centrifugation, washed with sterile water and prepared onto glass microscope slides with a coverslip. Live cells were imaged within 1 h of preparation. A Leica DMI6000 fluorescence microscope (Leica Microsystems, Buffalo Grove, IL, USA) with a 100× objective lens was used. Fluorescence filters included both GFP and mCherry (Semrock, GFP-4050B-LDKM-ZERO and mCherry-C LDMK-ZERO). The Leica Microsystems Application Suite AF software was used to capture images. All images within a single experimental trial were captured with identical exposure times. For clarity of presentation (and ease of visualizing cell morphology), the differential interference contrast (DIC) image contrast was adjusted prior to being merged with the mCherry channel. The fluorescence images were not altered except for cropping the image to illustrate a representative sampling of cells. Our analysis focused on the localization and pattern of GFP fluorescence within dividing cells and co-localization to mCherry (septin) signal. It did not address potential changes in cell morphology and/or the budding pattern of dividing cells. Finally, images may contain some of the boundary outside of the GFP/mCherry filter set; however, these areas typically did not contain any cells within the image and were on the periphery of the chosen image. All experiments were performed in at least triplicate.

## Results

### Bud3 truncations reveal domains required for efficient bud neck localization

**Fig. 1.**
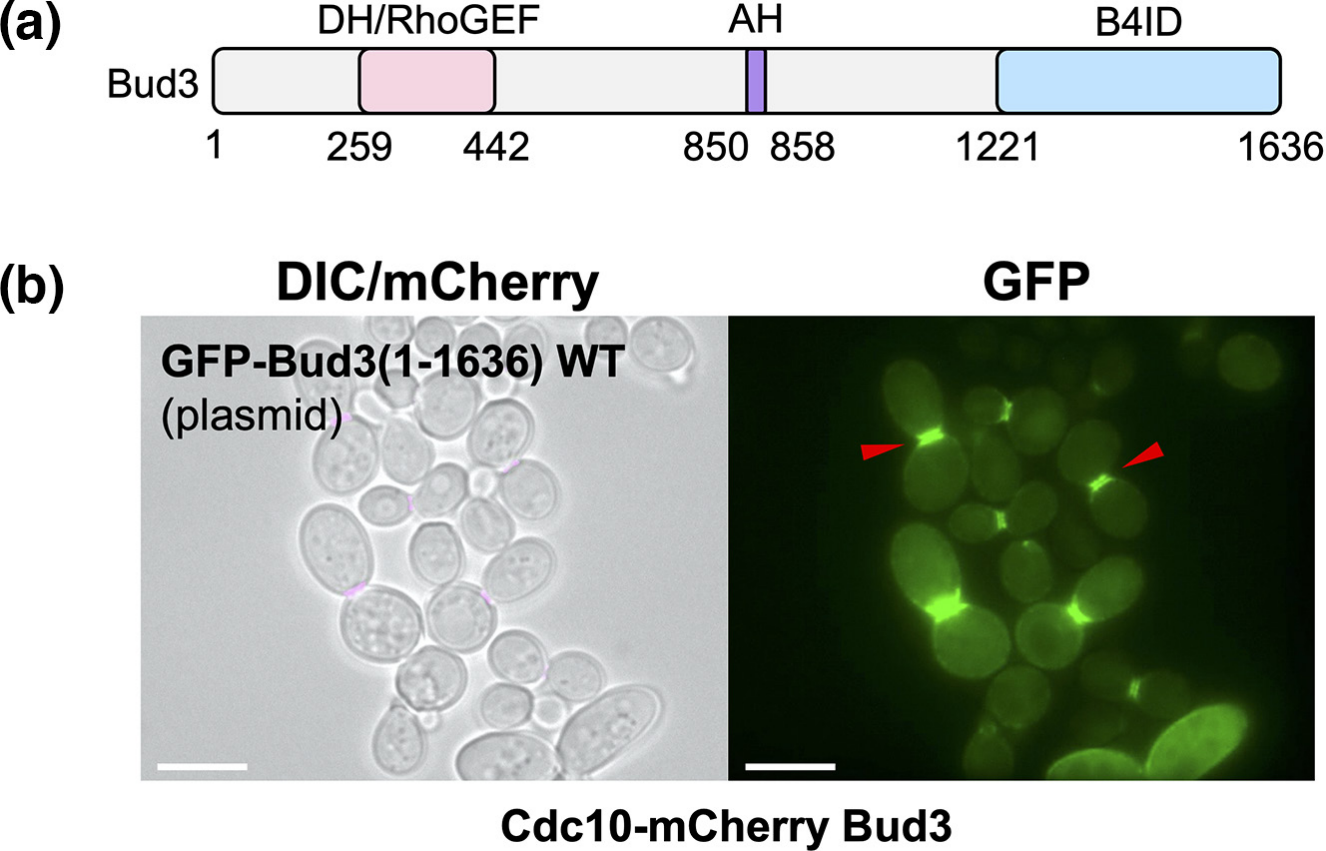
Bud3 localizes at the bud neck during cell division with the septin structure. (**a**) Primary structure of Bud3 protein; previously characterized features include the Dbl homology domain/Rho guanine nucleotide exchange factor (DH/RhoGEF), amphipathic helix (AH), Bud4-interacting domain (B4ID) (modelled after Ref. [27]). (**b**) Yeast strain GFY-42 expressing Cdc10-mCherry from the endogenous *CDC10* locus was transformed with a plasmid expressing GFP-tagged full-length Bud3 (pGF-IVL-1631). Of note, haploid yeast also contain a WT copy of untagged *BUD3* within the genome. Cells were incubated overnight in SD-LEU at 30 °C, back-diluted into YPD, grown for an additional 4 h at 30 °C, and visualized by fluorescence microscopy. The DIC and mCherry channels were merged; for clarity, the contrast of the DIC image was adjusted. Representative cells are presented. Scale bar, 7.5 µm. Red triangles denote the position of GFP signal at the bud neck in cells also displaying mCherry signal.

We began our investigation of cellular Bud3 localization and recruitment to the division site in yeast by testing expression of the full-length protein (Fig. 1a) *in vivo*. We expressed GFP-Bud3(1–1636) under control of the *CDC11* promoter (plasmid) in yeast containing the septin subunit Cdc10 tagged with mCherry (integrated) (Fig. 1b). In cells undergoing mitosis (displaying a prominent septin structure at the bud neck), GFP-Bud3(1–1636) co-localized with mCherry signal at the division site along with diffuse cytosolic signal and some weak GFP signal at the cell periphery that appeared as a weak continuous signal or small puncta (Fig. 1b). The detection of WT Bud3 protein at the plasma membrane is likely due to the experimental setup; our system included expression of GFP-Bud3 on a plasmid with a native copy of *BUD3* within the genome of haploid yeast. Our reasoning for analysing GFP-Bud3 (or various mutants or fusions) localization within a *BUD3-*containing cell was (i) to not require the Bud3 variant of interest to complement the function(s) of WT Bud3, (ii) to standardize the genetic background in which all experiments were being performed in terms of growth rate and cell morphology, (iii) to require Bud3 variants to ‘compete’ with the natural quantity of full-length untagged Bud3 also present in cells and (iv) to allow for the possibility of homotypic interaction between tagged and untagged WT Bud3 protein. Using these parameters, our goal was to perform a systematic analysis of the protein structure of Bud3 to identify domain(s) that were either necessary and/or sufficient for maintaining recruitment to the septin collar in dividing cells.

**Fig. 2.**
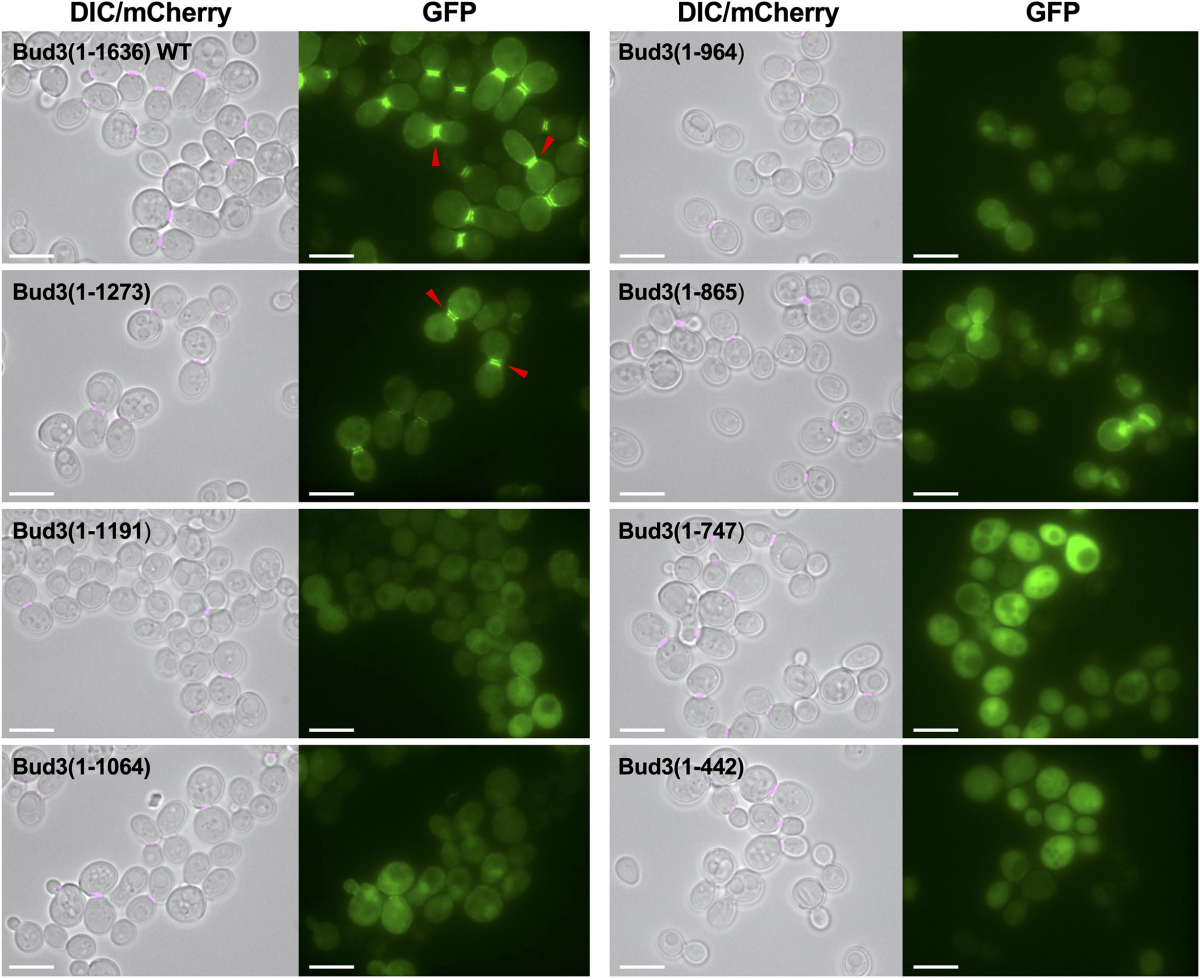
The subcellular localization of GFP-Bud3 fusions truncated at the C-terminus. Yeast containing an integrated Cdc10-mCherry construct (GFY-42) were transformed with plasmids (pGF-IVL-1631, pGF-IVL-11–124, pGF-IVL-11–121 and pGF-IVL-11–137 to pGF-IVL-11–141). Cells were visualized as in Fig. 1. The DIC and mCherry channels were merged for clarity. Scale bar, 7.5 µm. Red triangles denote the position of GFP signal at the bud neck of dividing yeast cells.

We began our analysis by testing GFP-Bud3 variants with increasing truncations at the C-terminus (Fig. 2) positioned within various previously defined structures across the full-length protein (Fig. 1a). Expression of GFP-Bud3(1–1273) still displayed GFP signal at the bud neck, albeit a weaker signal when compared to the WT control (Fig. 2). However, the localization pattern changed dramatically for GFP-Bud3(1–1191), which showed GFP signal within the cytosol and cell periphery and almost none concentrated at the bud neck; it was unclear if the faint fluorescence at or near the bud neck was due to recruitment to the septin structure or association with the membrane (Fig. 2). This same pattern was also observed for GFP-Bud3(1–1064), GFP-Bud3(1-964) and GFP-Bud3(1-865) (Fig. 2). Expression of GFP-Bud3(1-747) or GFP-Bud3(1-442) only resulted in cytosolic GFP signal; previous work [23] has demonstrated that a central AH domain (850-858) promotes membrane association for Bud3 and neither of these fragments contained this region (Fig. 2). Finally, a number of constructs displayed a cellular pattern that is likely to be the yeast nucleus undergoing division. Since this was not observed for WT Bud3 in our experiments, it was likely due to presentation of one or more nuclear localization signals (NLSs) that were not readily accessible in the full-length protein but became exposed within truncated variants. These data suggest that the bud neck localization of GFP-Bud3 depends on a domain defined by residues 1192–1273.

**Fig. 3.**
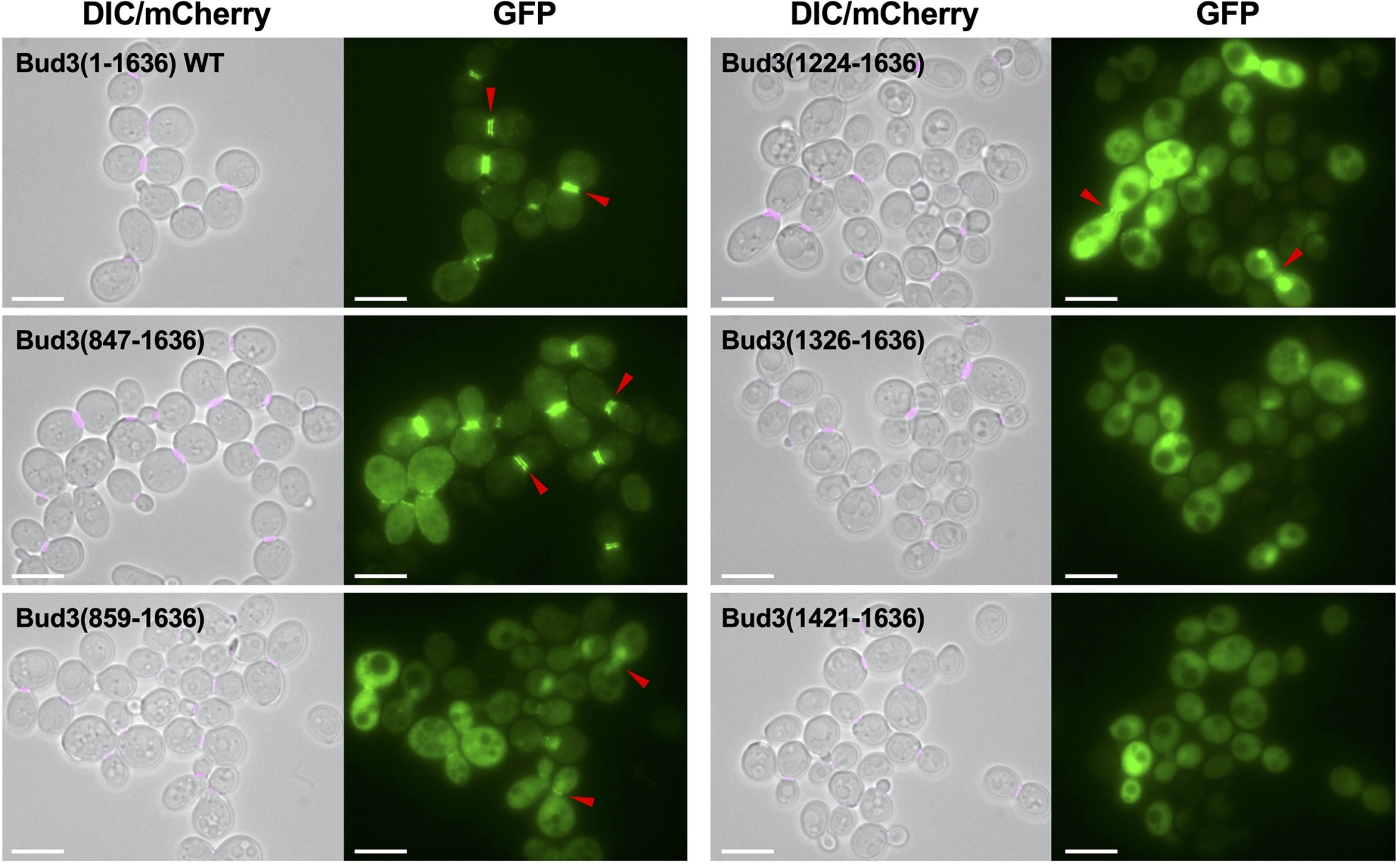
The subcellular localization of GFP-Bud3 fusions truncated at the N-terminus. Yeast containing an integrated Cdc10-mCherry construct (GFY-42) were transformed with plasmids (pGF-IVL-1631, pGF-IVL-11–6, pGF-IVL-11–12, pGF-IVL-11–15, pGF-IVL-11–18 and pGF-IVL-11–21) and were visualized as in Fig. 1. The DIC and mCherry channels were merged; scale bar, 7.5 µm. Red triangles denote the position of GFP signal at the bud neck of dividing yeast cells.

Next, we performed a complementary analysis by generating truncations at the N-terminus of Bud3 while still including GFP at the N-terminus of the fusion protein (Fig. 3). Removal of the first 846 amino acids within the GFP-Bud3(847–1636) construct had little overall effect on the localization pattern, which phenocopied the WT control (Fig. 3). However, extending the truncation beyond the central AH domain within GFP-Bud3(859–1636) caused a marked reduction in GFP at the bud neck (Fig. 3). Cells displayed a low but reproducible amount of fluorescence at the division site and loss of any signal at the cell periphery; this same pattern was also seen for the GFP-Bud3(1224–1636) construct (Fig. 3). Finally, expression of either GFP-Bud3(1326–1636) or GFP-Bud3(1421–1636) resulted in no GFP signal at the bud neck and only robust cytosolic localization (Fig. 3). Together, these experiments suggest a number of findings. First, the AH domain (residues 847–858, as defined by our construct) is essential for promoting robust bud neck localization of the C-terminal portion (residues 859–1636) of Bud3. Second, a small domain appears necessary for bud neck recruitment (albeit, very weak) within the region of 1224–1325, although this may also include surrounding residues. Third, the extreme C-terminal domain (residues 1326–1636) is not sufficient to promote bud neck localization when expressed alone. Comparison of the data from the C-terminal (Fig. 2) and N-terminal (Fig. 3) truncations reveals that a likely candidate for a bud neck localization domain within Bud3 is centred around residues 1192–1325.

### Bud3 fusion proteins reveal a conserved motif sufficient for bud neck localization

**Fig. 4.**
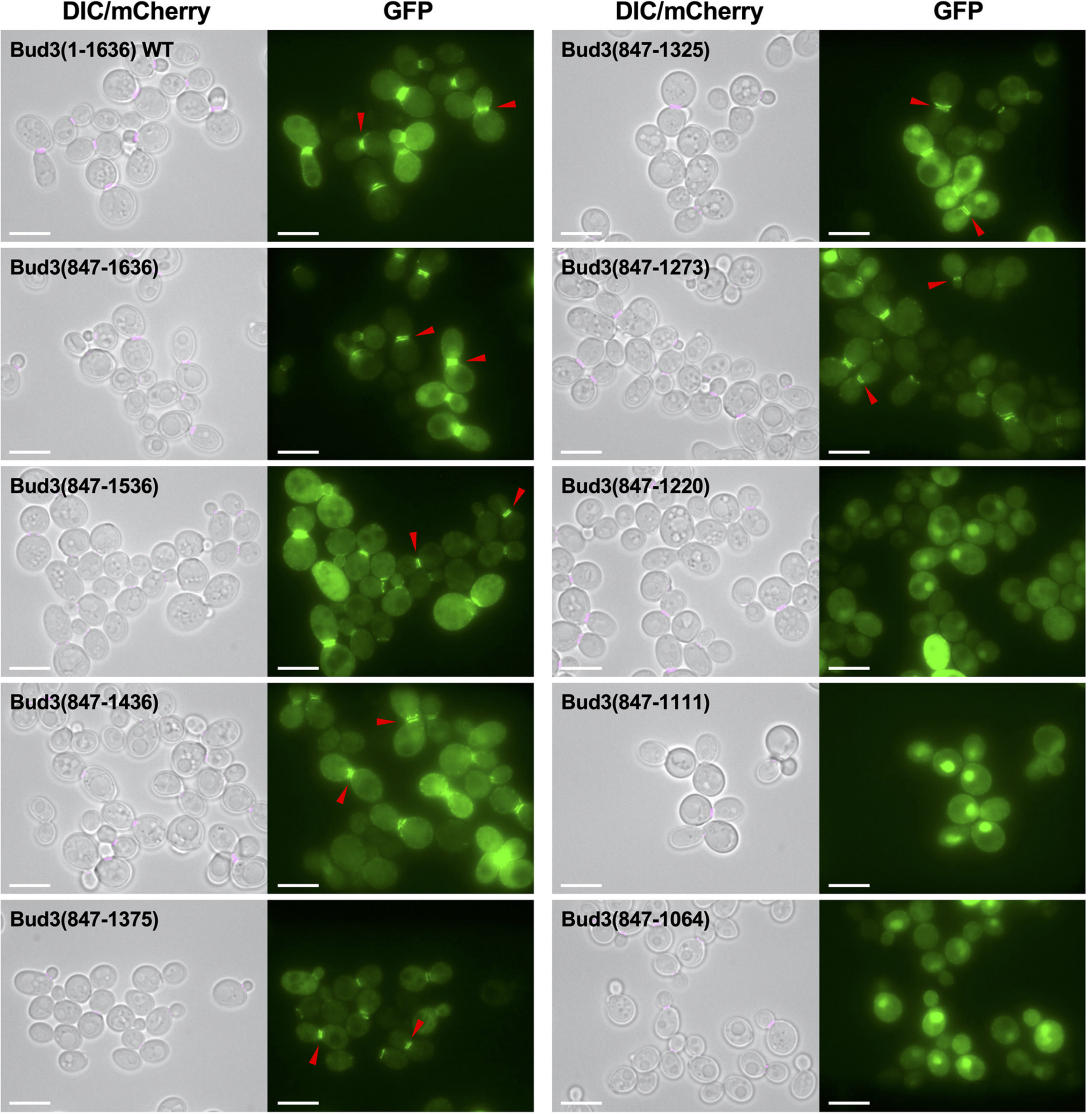
A minimal continuous Bud3 fragment sufficient for bud neck localization *in vivo*. Yeast (GFY-42) were transformed with plasmids (pGF-IVL-1631, pGF-IVL-11–1 through pGF-IVL-11–6 and pGF-IVL-11–31 through pGF-IVL-11–33) and visualized as in Fig. 1. The DIC and mCherry channels were merged. Scale bar, 7.5 µm. Red triangles denote the position of GFP signal at the bud neck of dividing yeast cells.

Since removal of the first 846 amino acids had little effect on the localization pattern of GFP-Bud3 *in vivo*, we generated a set of C-terminal truncations while maintaining 847 as the first amino acid of Bud3 to ensure inclusion of the AH domain (Fig. 4). We observed that deleting progressively larger fragments from the C-terminus did not cause any change in localization phenotype for GFP-Bud3(847–1536), GFP-Bud3(847–1436), GFP-Bud3(847–1375), GFP-Bud3(847–1325) and GFP-Bud3(847–1273) (Fig. 4). These constructs all displayed similar GFP patterns at the bud neck. However, expression of GFP-Bud3(847–1220) or smaller fragments such as GFP-Bud3(847–1111) or GFP-Bud3(847–1064) displayed a total loss of bud neck localization (Fig. 4). These data suggest that critical localization information resides within (or near) residues 1221–1273. Moreover, we conclude that residues 1274–1325 do not provide a strong contribution to this cellular pattern, at least in the context of this truncated Bud3 construct.

**Fig. 5.**
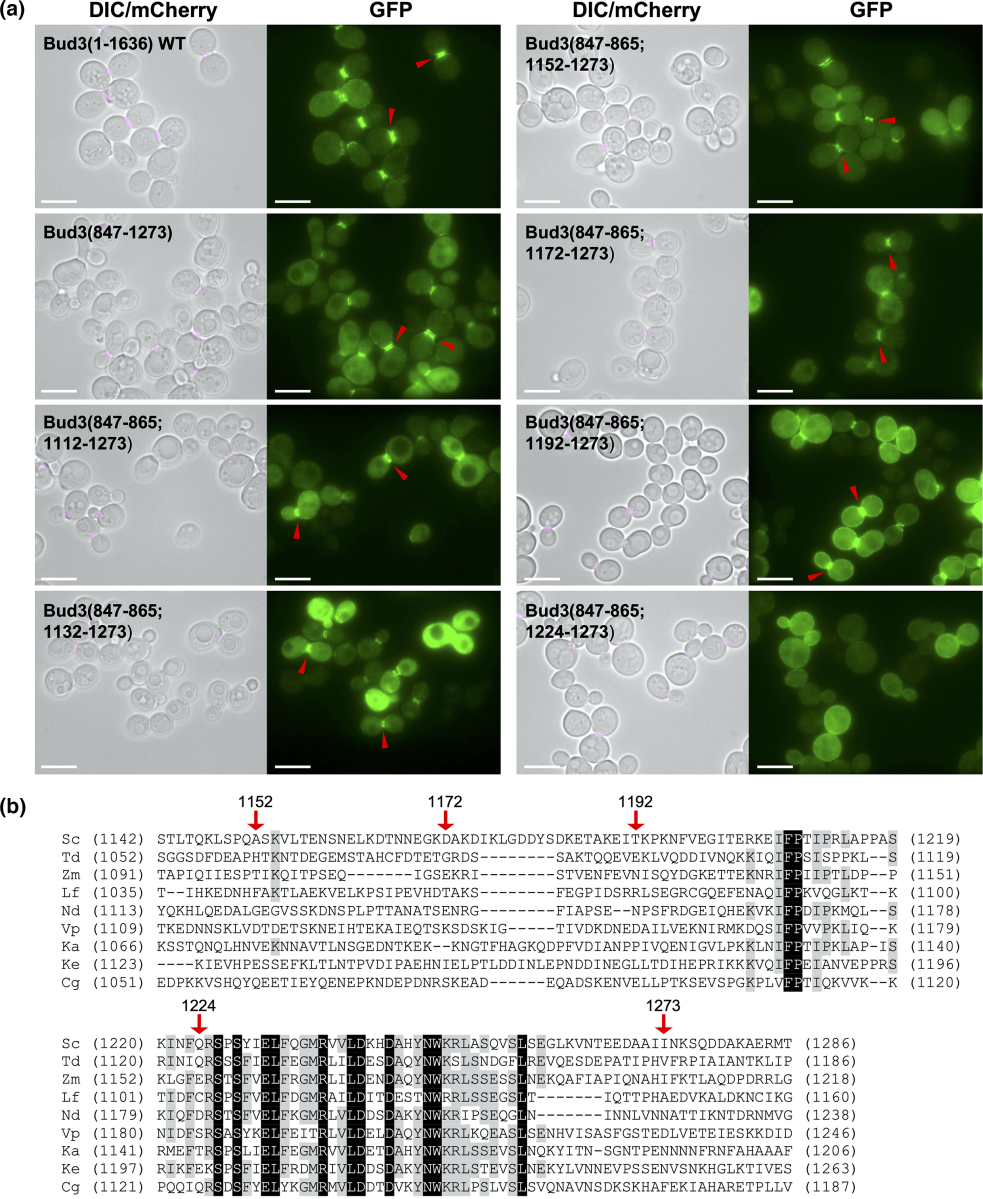
Direct fusions between the central amphipathic helix and C-terminal fragments of Bud3 reveal a minimal construct for bud neck localization. (**a**) Yeast (GFY-42) were transformed with plasmids (pGF-IVL-1631, pGF-IVL-11–31, pGF-IVL-11–41, pGF-IVL-11–69 and pGF-IVL-11–76 through pGF-IVL-11–79) and visualized as in Fig. 1. The DIC and mCherry channels were merged. Scale bar, 7.5 µm. Red triangles denote the position of GFP signal at the bud neck of dividing yeast cells. (**b**) Protein sequence alignment of putative Bud3 orthologues from related fungal species. Full-length Bud3(1–1636) from *S. cerevisiae* was used as a query sequence to identify similar proteins within the fungal kingdom using blast (NCBI). Full-length proteins from eight species (Fig. **S2**) were aligned against *S. cerevisiae* Bud3 using clustal-W [31]. The alignment for residues 1142–1286 (numbering from budding yeast Bud3) is displayed. Residues identical across all nine species are coloured white against a black background; residues identical across at least five of the nine species are coloured black against a grey background. Red arrows illustrate specific residue positions in *S. cerevisiae* Bud3.

Thus far, our analysis narrowed the focus on Bud3 residues between 1192 and 1273 as necessary in the context of specific truncations from either terminus. Therefore, we built a series of fusion proteins that included the AH domain (847-865) paired with smaller fragments *in trans* that all ended at position 1273 (Fig. 5). Our primary interest was to test whether residues immediately proximal to position 1192 might also contribute to a localization signal. Expression of GFP-Bud3(847–865; 1112–1273), GFP-Bud3(847–865; 1132–1273), GFP-Bud3(847–865; 1152–1273) and GFP-Bud3(847–865; 1172–1273) all displayed a similar pattern of GFP signal at the bud neck in dividing cells (Fig. 5). However, there was a noticeable shift in the overall localization for GFP-Bud3(847–865; 1192–1273) with more robust fluorescence present at the cell periphery (Fig. 5). Finally, shortening the construct within GFP-Bud3(847–865; 1224–1273) resulted in a loss of bud neck localization (Fig. 5).

To investigate whether there was an explanation for the difference in phenotype between Bud3 fusions that included fragments beginning at 1172, 1192, or 1224, we performed a search for other Bud3 orthologues in related fungal species (Figs 5b and S2). An alignment of proteins similar to Bud3 revealed several unique features when examining the *S. cerevisae* region from 1142 to 1286 (Fig. 5b). First, the presence of a conserved motif appears within yeast Bud3 at residues 1207–1260 (Fig. 5b). This region includes 13 amino acids that are identical across 9/9 species and 20 additional residues that are conserved in at least 5/9 fungi (Fig. 5b). This is in stark contrast to the surrounding regions (1142–1206 or 1261–1286), where there is almost no conservation of amino acids (Fig. 5b). Given that the GFP-Bud3(847–865; 1192–1273) displayed bud neck localization, whereas the GFP-Bud3(847–865; 1224–1273) construct did not, it may be that (i) the signal requires a minimal number of essential residues within the motif and/or (ii) there needs to be adequate physical distance in the form of a linker sequence extending between the AH domain contacting the membrane and bud neck association. Our data illustrate two separate reductions in bud neck localization as the Bud3 domain was shortened from position 1112 to 1224 (Fig. 5). The GFP–Bud3(847–865; 1172–1273) construct displayed less GFP signal at the cell periphery compared to GFP-Bud3(847–865; 1192–1273); we suspect that this may be due to decreased binding and/or suboptimal positioning of the C-terminal fragment to the bud neck (Fig. 5). Second, we observed total loss of recruitment to the division site once the fragment was shortened to residue 1224 and this may be due to proximity to the evolutionarily conserved motif present between residues 1207–1260 (Fig. 5b).

**Fig. 6.**
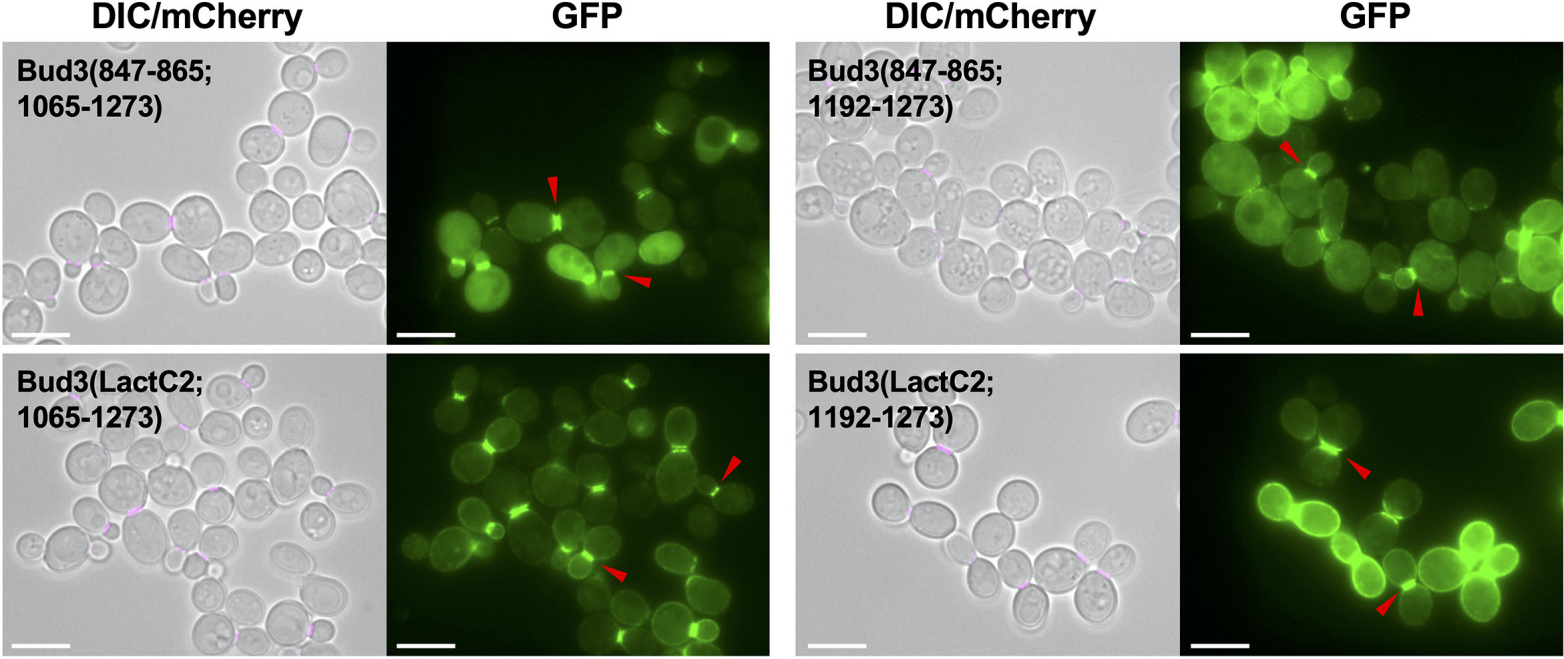
Replacement of the Bud3 amphipathic helix with LactC2 domain. Yeast (GFY-42) containing plasmids (pGF-IVL-11–68, pGF-IVL-11–79, pGF-IVL-11–107 and pGF-IVL-11–110) were cultured and visualized as in Fig. 1. The DIC and mCherry channels were merged. Scale bar, 7.5 µm. Red triangles denote the position of GFP signal at the bud neck of dividing yeast cells.

To examine whether the conserved Bud3 motif was sufficient to promote bud neck localization in an independent context, we generated additional fusions using a separate protein domain that associates with membranes (Fig. 6). We replaced the AH (847–865) domain with the 158-residue C2 domain from bovine lactadherin (LactC2), which binds to phosphatidylserine on the PM [29, 30]. Compared to GFP-Bud3(847–865; 1065–1273), the GFP-Bud3(LactC2; 1065–1273) fusion displayed robust localization to the bud neck as well as an increased level at the plasma membrane (Fig. 6). This effect was also evident when examining GFP-Bud3(847–865; 1192–1273) against GFP-Bud3(LactC2; 1192–1273) (Fig. 6). In both cases, these additional constructs illustrated that (i) membrane association, rather than the identity of the AH domain residues, was critical to promote bud neck localization and (ii) a non-native protein domain separating GFP from the central domain of Bud3 (1065–1273 or 1192–1273) still allowed for robust fluorescence signal at the division site, suggesting that membrane association and the bud neck signal work in concert but are separable.

## Discussion

Using a series of truncations and fusion proteins, our study has defined two small domains that coordinate *in trans* to promote optimal localization of the Bud3 protein to the bud neck. This includes the central AH domain (847–865) and a fragment at the C-terminus (1172–1273) with a small conserved region at residues 1207–1260. A previous study reported that three separate regions spanning the full-length Bud3 (1–946, 674–1220 and 1221–1636) could each promote bud neck localization [23]. A number of critical differences exist with our experimental approach, including the choice of the *CDC11* promoter driving expression of GFP-Bud3 as opposed to *MET25*, as well as the use of a *BUD3*-expressing WT background rather than a *bud3∆* strain. It remains possible that the combination of overexpression of Bud3 fragments (such as 1–946 or 674–1220) could localize to the division site in the absence of competing WT Bud3. If any such targeting signal(s) exists within the N-terminal region of Bud3, it is extremely weak compared to the C-terminal information we have identified in this study; along these lines, the previous report observed that localization of Bud3(1–946) was markedly lower than that of WT protein [23]. Furthermore, numerous studies have suggested both Bud4-dependent and Bud4-independent mechanisms for bud neck recruitment of Bud3 [23, 27]; therefore, it is highly likely that numerous targeting signals exist within the protein.

Interestingly, previous work on Bud3 has provided limited information on the nature of the bud neck targeting domain within the C-terminal region defined by residues 1221–1636, an arbitrarily designated fragment from a previous study [23]. Yeast two-hybrid experiments illustrated that Bud3(948–1466) and Bud3(1221–1614) were both able to interact with the TD2 domain of Bud4 (residues 1067–1447) [25]. The conclusion, given the overlap between both Bud3 fragments, was that the region of 1221–1466 was sufficient to interact with the C-terminus of Bud4, although this exact fragment was not experimentally tested [25]. Furthermore, the ability of Bud3(1221–1636) to localize to the bud neck was compromised in *bud4∆* and *bud3∆ bud4∆* strains, supporting a model where this region of Bud3 requires Bud4 for recruitment [25]. Other groups have also demonstrated that loss of the C-terminal region of Bud3 (1221–1636) causes a (partial) loss of recruitment to the division site similar to a loss of Bud4 [27]. Our findings suggest that a much smaller domain within this region (1221–1636) may be sufficient for bud neck recruitment *in vivo* even in the presence of endogenous Bud3. We propose that the domain may coincidently begin near residue 1221 yet only extend to residue 1273 (smaller than the 1466 proposed from yeast two-hybrid data). From our Bud3 fusion constructs, we observed improved bud neck localization when the minimal fragment was extended from 1192 to 1172. Given that none of these residues were conserved even between closely related species, it seems plausible that this represents physical separation from the fused AH domain to potentially allow proper association at the bud neck (and possibly physical interaction with the C-terminus of Bud4). Future biochemical interaction studies (with the TD2 domain of Bud4 or septins) and/or a mutational analysis of this smaller fragment and the highly conserved residues found near position 1221 will provide additional information about this targeting region within Bud3.

Our study also highlighted that, like other large multi-domain proteins localized to the septin collar and bud neck, a membrane-association domain acts to optimize and promote efficient localization *in vivo*. Our previous work demonstrated that Hsl1, a septin-interacting kinase responsible for the G2/M checkpoint, utilizes a central septin-binding domain with a C-terminal membrane-binding KA1 domain [13]. Here, we illustrated bud neck recruitment of the C-terminal domain of Bud3 when it was combined with the central AH domain either *in cis* or *in trans*; additionally, use of the non-native membrane-associating LactC2 domain also had the same effect.

Similar to previous findings that deletion of the Bud3(1221–1636) Bud4-interacting domain caused a reduction (but not total loss) of bud neck targeting [27], we also found that a full-length Bud3 construct lacking only the central 1192–1273 domain was still able to localize to the division site, albeit with much weaker signal (our unpublished data). Additional experimentation will be required to identify the potential Bud4-independent mechanisms allowing for bud neck recruitment using variants of Bud3 lacking 1192–1273 (or 1221–1636) and/or in *bud4∆* cells. It would be of interest to elucidate whether region(s) of Bud3 can associate directly with the septin superstructure or one or more of the many other proteins targeted to the bud neck during cell division [3].

### Reagent availability statement

Yeast and/or plasmids will be made available for educational or research purposes upon reasonable request.

## Supplementary Data

Supplementary material 1
