# Supplementary material for: Characterization of Bud3 domains sufficient for bud neck targeting in S. cerevisiae
Source: Access Microbiol. 2022 Mar 24;4(3):000341. doi: 10.1099/acmi.0.000341 (PMC9175976; doi:10.1099/acmi.0.000341)
Supplement: Supplementary material 1 [file acmi-4-0341-s001.pdf]

SUPPLEMENTAL TABLES and FIGURES for

Characterization of Bud3 domains sufficient for bud neck targeting in *S. cerevisiae*

Madison N. Schrock[1,2]*, Yao Yan[1]*, Megan E. Goeckel[1,3], Erianna M. Basgall[1,4], Isabel C. Lewis[1,5], Katherine G. Leonard[1,6], Megan Halloran[1,7], and Gregory C. Finnigan[1†]

[1]Department of Biochemistry and Molecular Biophysics,

Kansas State University, 141 Chalmers Hall, Manhattan, KS 66506 USA

[2]Current Address: School of Biological Sciences, University of Utah, Salt Lake City, UT, 84112 USA

[3]Current Address: Department of Cell Biology and Physiology, Washington University in St. Louis, School of Medicine, St. Louis, MO, 63110 USA

[4]Current Address: Department of Neurobiology, School of Medicine, University of Utah, Salt Lake City, UT, 84112 USA

[5]Current Address: School of Medicine, University of Texas Medical Branch, Galveston, TX, 77555 USA

[6]Current Address: Memorial Sloan Kettering Cancer Center, New York, NY, 10065 USA

[7]Current Address: Department of Psychology, University of Kentucky, Lexington, KY, 40506 USA

*These authors contributed equally to this work.

Keywords: Bud3, septins, bud neck, fluorescence microscopy

[†]Correspondence to:

Gregory C. Finnigan

Dept. of Biochemistry & Molecular Biophysics

Kansas State University

141 Chalmers Hall, 1711 Claflin Rd.

Manhattan, KS 66506 USA

Phone: (785) 532-6939; FAX; (785) 532-7278;

E-mail: gfinnigan@ksu.edu

Supplemental Table S1. Yeast and DNA plasmids used in this study.

**Strain**	**Genotype**	**Reference**
GFY-42[a,b]	*MAT***a** *cdc10Δ::S.c.CDC10::mCherry::ADH1(t)::S.p.HIS5*	[1]
**Plasmid**	**Description**	**Reference**
pRS315	*CEN*, *LEU2*	[2]
pGF-IVL-1631[c,d,e]	pRS315; *prCDC11::GFP::Bud3(1-1636)::ADH1(t)::Hyg*^{R}	This study
pGF-IVL-11-1	pRS315; *prCDC11::GFP::Bud3(847-1064)::ADH1(t)::Hyg*^{R}	This study
pGF-IVL-11-2	pRS315; *prCDC11::GFP::Bud3(847-1111)::ADH1(t)::Hyg*^{R}	This study
pGF-IVL-11-3	pRS315; *prCDC11::GFP::Bud3(847-1220)::ADH1(t)::Hyg*^{R}	This study
pGF-IVL-11-4	pRS315; *prCDC11::GFP::Bud3(847-1436)::ADH1(t)::Hyg*^{R}	This study
pGF-IVL-11-5	pRS315; *prCDC11::GFP::Bud3(847-1536)::ADH1(t)::Hyg*^{R}	This study
pGF-IVL-11-6	pRS315; *prCDC11::GFP::Bud3(847-1636)::ADH1(t)::Hyg*^{R}	This study
pGF-IVL-11-12	pRS315; *prCDC11::GFP::Bud3(859-1636)::ADH1(t)::Hyg*^{R}	This study
pGF-IVL-11-15	pRS315; *prCDC11::GFP::Bud3(1224-1636)::ADH1(t)::Hyg*^{R}	This study
pGF-IVL-11-18	pRS315; *prCDC11::GFP::Bud3(1326-1636)::ADH1(t)::Hyg*^{R}	This study
pGF-IVL-11-21	pRS315; *prCDC11::GFP::Bud3(1421-1636)::ADH1(t)::Hyg*^{R}	This study
pGF-IVL-11-31	pRS315; *prCDC11::GFP::Bud3(847-1273)::ADH1(t)::Hyg*^{R}	This study
pGF-IVL-11-32	pRS315; *prCDC11::GFP::Bud3(847-1325)::ADH1(t)::Hyg*^{R}	This study
pGF-IVL-11-33	pRS315; *prCDC11::GFP::Bud3(847-1375)::ADH1(t)::Hyg*^{R}	This study
pGF-IVL-11-42	pRS315; *prCDC11::GFP::Bud3(847-865; 1224-1273)::ADH1(t)::Hyg*^{R}	This study

pGF-IVL-11-68	pRS315; *prCDC11::GFP::Bud3(847-865; 1065-1273)::ADH1(t)::Hyg*^{R}	This study
pGF-IVL-11-69	pRS315; *prCDC11::GFP::Bud3(847-865; 1112-1273)::ADH1(t)::Hyg*^{R}	This study
pGF-IVL-11-76	pRS315; *prCDC11::GFP::Bud3(847-865; 1132-1273)::ADH1(t)::Hyg*^{R}	This study
pGF-IVL-11-77	pRS315; *prCDC11::GFP::Bud3(847-865; 1152-1273)::ADH1(t)::Hyg*^{R}	This study
pGF-IVL-11-78	pRS315; *prCDC11::GFP::Bud3(847-865; 1172-1273)::ADH1(t)::Hyg*^{R}	This study
pGF-IVL-11-79	pRS315; *prCDC11::GFP::Bud3(847-865; 1192-1273)::ADH1(t)::Hyg*^{R}	This study
pGF-IVL-11-107[f]	pRS315; *prCDC11::GFP::LactC2(1-158)::Bud3(1065-1273)::ADH1(t)::Hyg*^{R}	This study
pGF-IVL-11-110	pRS315; *prCDC11::GFP::LactC2(1-158)::Bud3(1192-1273)::ADH1(t)::Hyg*^{R}	This study
pGF-IVL-11-121[g]	pRS315; *prCDC11::GFP::Bud3(1-1064)::ADH1(t)::Kan*^{R}	This study
pGF-IVL-11-124	pRS315; *prCDC11::GFP::Bud3(1-1191)::ADH1(t)::Kan*^{R}	This study
pGF-IVL-11-137	pRS315; *prCDC11::GFP::Bud3(1-442)::ADH1(t)::Hyg*^{R}	This study
pGF-IVL-11-138	pRS315; *prCDC11::GFP::Bud3(1-747)::ADH1(t)::Hyg*^{R}	This study
pGF-IVL-11-139	pRS315; *prCDC11::GFP::Bud3(1-865)::ADH1(t)::Hyg*^{R}	This study
pGF-IVL-11-140	pRS315; *prCDC11::GFP::Bud3(1-964)::ADH1(t)::Hyg*^{R}	This study
pGF-IVL-11-141	pRS315; *prCDC11::GFP::Bud3(1-1273)::ADH1(t)::Hyg*^{R}	This study

[a]Abbreviations of the fungal genus and species are included for the *CDC10* and *HIS5* genes: *S.c., Saccharomyces cerevisiae; S.p. Schizosaccharomyces pombe.*
[b]The mCherry sequence used:
VSKGEEDNMAIIKEFMRFKVHMEGSVNGHEFEIEGEGEGRPYEGTQTAKLKVTKGGPLP
FAWDILSPQFMYGSKAYVKHPADIPDYLKLSFPEGFKWERVMNFEDGGVVTVTQDSSL
QDGEFIYKVKLRGTNFPSDGPVMQKKTMGWEASSERMYPEDGALKGEIKQRLKLKDG
GHYDAEVKTTYKAKKPVQLPGAYNVNIKLDITSHNEDYTIVEQYERAEGRHSTGGMDE
LYK.
[c]The GFP sequence used:
MGRRIPGLINSKGEELFTGVVPILVELDGDVNGHKFSVSGEGEGDATYGKLTLKFICTTG
KLPVPWPTLVTTLTYGVQCFSRYPDHMKRHDFFKSAMPEGYVQERTIFFKDDGNYKTR
AEVKFEGDTLVNRIELKGIDFKEDGNILGHKLEYNYNSHNVYIMADKQKNGIKVNFKIR
HNIEDGSVQLADHYQQNTPIGDGPVLLPDNHYLSTQSALSKDPNEKRDHMVLLEFVTAA
GITHGMDELYK.
[d]The commonly used *prMX* and *MX(t)* sequences were included flanking all drug resistance cassettes (such as Hyg^R).
[e]The strategy for plasmid construction included use of a parental vector built on pRS315 that included pr*CDC11*-GFP-[*SpeI*]-*ADH1*(t)-Hyg^R. Linearization was achieved by digestion of the unique *SpeI* restriction site between the GFP and *ADH1*(t) sequences. Digested plasmid was then co-transformed with amplified *BUD3* PCR(s) with flanking homology to upstream GFP sequence and downstream *ADH1*(t) sequence into yeast for *in vivo* plasmid assembly.
[f]The *Bos taurus* lactadherin C2 (Lact-C2) domain sequence [3,4] was fused to each construct at the 3' end of the GFP gene. The numbering scheme for Lact-C2 refers to the specific domain and not the full-length lactadherin protein. The amino acid sequence of the 158 residue domain:
CTEPLGLKDNTIPNKQITASSYYKTWGLSAFSWFPYYARLDNQGKFNAWTAQTNSASE
WLQIDLGSQKRVTGIITQGARDFGHIQYVAAYRVAYGDDGVTWTEYKDPGASESKIFPG
NMDNNSHKKNIFETPFQARFVRIQPVAWHNRITLRVELLGC.
[g]For constructs tagged with Kan^R (rather than Hyg^R), a similar cloning strategy was employed. Rather than use of a parental construct already containing GFP and the drug cassette, a plasmid with the *CDC11* promoter was linearized and used for *in vivo* assembly. The included GFP, *BUD3* fragments, *ADH1*(t) and Kan^R were included on amplified PCR fragments.

Supplemental Figure S1. DNA Sequences used in this study. The universal *S. pombe HIS5*, Kan^R, Hyg^R MX-based cassettes [5] were used following the *ADH1* terminator sequence for integrated or plasmid-born constructs.

```
prCDC10-CDC10-mCherry-ADH1(t)
(from strain GFY-42)

GTATTGGCTCTCTTATAATGGCCGCATGATGGGCACTTATTCAAATGGTGAATCATCTTCAATTGCTTCTTACCTGG
ACCGTAAAGTTTTTGCCTTTTTTTTTGGTGTGATACTTTTTTTTTAGGAACTGCTAGTAGAATACCATTATTAGAGA
AAAAATCTTTATCTTCAGTGTCCTTATGATCTTGTTGTAGCTTTTCTCTCAACCAACGGTGGATAGATCCCAGTTGT
TTTTGGCCTGGAATCTGCAGTGAAGGATTTCCTAGCGGTACCAACCACCCAATTGCAGTGGTAGGCACAATTTTGTG
AAATGCTAATTGTTTACCAAAAATCAAAGAATTCATAGTTTGTTTGCTGATATTCCCTGGTGTTTTTGTCTCCAAGT
TAAGGTATTATTTGGGAACATTGTGCCTTATATTAAGATTTGCTTTCTGAATGCTTGCGGAAATAATCGTATGATAC
GATGAGATTTCCTCGAGAGACCACAAATAGATCAGAATCGCTGAATAGAATATATGTAAACATGTATCAGTAATACT
TAACTTTTTTCAGGCAAAGACAAGAAAATACAAGGCCAAGCCCCACGGTTACTACAAGCACTCTATAAATATATTAA
TGGATCCTCTCAGCTCAGTACAGCCTGCTTCTTATGTTGGTTTTGATACCATCACGAATCAGATCGAACATCGTCTG
TTGAAGAAAGGTTTTCAATTTAATATAATGGTTGTTGGCCAATCCGGATTGGGTAAAAGTACTCTAATAAATACGTT
ATTTGCCTCACATTTGATTGATTCTGCTACTGGTGATGATATTTCTGCCCTGCCTGTTACAAAAACAACTGAAATGA
AAATTTCTACTCATACTCTTGTGGAGGACCGCGTTCGCTTGAATATTAATGTTATAGATACACCTGGATTTGGTGAC
TTTATTGACAATTCTAAAGCTTGGGAGCCTATTGTGAAGTACATTAAGGAACAACATTCTCAATACTTACGTAAAGA
ATTGACAGCCCAACGTGAAAGGTTTATTACTGATACAAGAGTTCATGCAATTCTTTATTTCCTGCAACCAAATGGAA
AGGAGTTGAGCCGCCTTGACGTTGAAGCCTTGAAAAGATTGACAGAAATAGCAAATGTTATACCAGTTATTGGCAAG
TCGGATACATTGACTTTAGATGAAAGAACGGAGTTTAGGGAGCTTATTCAAAATGAATTCGAAAAATACAATTTCAA
GATTTATCCTTATGATTCGGAAGAACTAACTGACGAGGAATTAGAACTAAACAGAAGTGTTAGATCTATCATTCCGT
TTGCAGTGGTTGGTTCTGAGAATGAGATTGAAATAAACGGTGAAACCTTCAGGGGAAGAAAAACTCGTTGGAGCGCT
ATTAATGTTGAGGATATCAACCAGTGTGATTTTGTATATTTAAGGGAATTTTTGATTCGAACTCATCTCCAAGACTT
AATCGAAACAACTTCCTACATTCATTATGAAGGGTTCAGAGCAAGACAATTAATTGCCTTGAAAGAAAATGCGAATA
GTCGTTCCTCAGCTCATATGTCTAGCAACGCCATTCAACGTGTGAGCAAGGGCGAGGAGGATAACATGGCCATCATC
AAGGAGTTCATGCGCTTCAAGGTGCACATGGAGGGCTCCGTGAACGGCCACGAGTTCGAGATCGAGGGCGAGGGCGA
GGGCCGCCCCTACGAGGGCACCCAGACCGCCAAGCTGAAGGTGACCAAGGGTGGCCCCCTGCCCTTCGCCTGGGACA
TCCTGTCCCCTCAGTTCATGTACGGCTCCAAGGCCTACGTGAAGCACCCCGCCGACATCCCCGACTACTTGAAGCTG
TCCTTCCCCGAGGGCTTCAAGTGGGAGCGCGTGATGAACTTCGAGGACGGCGGCGTGGTGACCGTGACCCAGGACTC
CTCCCTGCAGGACGGCGAGTTCATCTACAAGGTGAAGCTGCGCGGCACCAACTTCCCCTCCGACGGCCCCGTAATGC
AGAAGAAGACCATGGGCTGGGAGGCCTCCTCCGAGCGGATGTACCCCGAGGACGGCGCCCTGAAGGGCGAGATCAAG
CAGAGGCTGAAGCTGAAGGACGGCGGCCACTACGACGCTGAGGTCAAGACCACCTACAAGGCCAAGAAGCCCGTGCA
GCTGCCCGGCGCCTACAACGTCAACATCAAGTTGGACATCACCTCCCACAACGAGGACTACACCATCGTGGAACAGT
ACGAACGCGCCGAGGGCCGCCACTCCACCGGCGGCATGGACGAGCTGTACAAGTAAGGCGCGCCACTTCTAAATAAG
CGAATTTCTTATGATTTATGATTTTTATTATTAAATAAGTTATAAAAAAAATAAGTGTATACAAATTTTAAAGTGAC
TCTTAGGTTTTAAAACGAAAATTCTTATTCTTGAGTAACTCTTTCCTGTAGGTCAGGTTGCTTTCTCAGGTATAGTA
TGAGGTCGCTCTTATTGACCACACCTCTACCGGCAGATCCGCTAGGGATAACAGGGTAATAT

prCDC11-GFP-Bud3(1-1636)-ADH1(t):
(from plasmid pGF-IVL-1636)

CCATGTATTTACTGACTTTGCAAAAGTCCAAATATCTGTAAATTAACATGCTATTATAAATATATATATATATATAT
ATTTGAATTTTCTATTTTTTCATCTACAAAAGCAGGTTATAGCTCCGTTAAACCCTTTGCTCATTGAATTGTCCTTC
ACGGTTTTTTGGTGGAAAACACAACATGGAACATAACATTTAAACATCGTTCTCAATCCATTTCATCGCAGTAACAT
ATTTGGTCGGGCACATACGGACACGTATATGTTATACAGATAAATATAGCTTAAATATGGCTATATTTACACAACAC
AATGCTCGTGGGTCGTTACCCGACCATGCAGGATCGCCAGTATTCTTTATTTTTCCGCCTTCTAACATAAAGAAAAA
TAAACAAAAAAGTATTTGATCGAAAAGTAAAATAGGTAGACACCACGTATTGGCGACCCGATCTGGAGCCGTTTAGA
AAGTCAATCATCACAAGGCCTAAAGTTGCTAACCACCAGCCATGGGTCGACGGATCCCCGGGTTAATTAACAGTAAA
GGAGAAGAACTTTTCACTGGAGTTGTCCCAATTCTTGTTGAATTAGATGGTGATGTTAATGGGCACAAATTTTCTGT
CAGTGGGGAGGGTGAAGGTGATGCAACATACGGAAAACTTACCCTTAAATTTATTTGCACTACTGGAAAACTACCTG
```

TTCCATGGCCAACACTTGTCACTACTTTGACTTATGGTGTTCAATGCTTTTCAAGATACCCAGATCATATGAAACGG
CATGACTTTTTCAAGAGTGCCATGCCCGAAGGTTATGTACAGGAAAGAACTATATTTTTCAAAGATGACGGGAACTA
CAAGACACGTGCTGAAGTCAAGTTTGAAGGTGATACCCTTGTTAATAGAATCGAGTTAAAAGGTATTGATTTTAAAG
AAGATGGAAACATTCTTGGACACAAATTGGAATACAACTATAACTCACACAATGTATACATCATGGCAGACAAACAA
AAGAATGGAATCAAAGTTAACTTCAAAATTAGACACAACATTGAAGATGGAAGCGTTCAACTAGCAGACCATTATCA
ACAAAATACTCCAATTGGCGATGGCCCTGTCCTTTTACCAGACAACCATTACCTGTCCACACAATCTGCCCTTTCGA
AAGATCCCAACGAAAAGAGAGACCACATGGTCCTTCTTGAGTTTGTAACAGCTGCTGGGATTACACATGGCATGGAT
GAACTATACAAAATGGAGAAAGACCTGTCGTCTCTTTACTCTGAAAAGAAAGACAAAGAGAACGATGAAACCTTATT
TAACATCAAACTATCCAAATCTGTTGTCGAGACCACACCGCTAAATGGTCATTCATTGTTTGATGATGATAAATCAC
TTTCAGACTGGACGGATAATGTGTTCACTCAATCAGTATTCTATCACGGGTCAGATGACTTGATATGGGGGAAGTTC
TTTGTCTGCGTGTACAAGTCCCCCAACAGCAATAAGTTGAACGCTATAATATTCGACAAATTAGGAACATCATGCTT
CGAATCCGTCGATATATCTTCCAACTCGCAATACTATCCGGCCATTGAGAATTTGAGTCCAAGTGATCAGGAAAGCA
ATGTTAAGAAATGCATTGCTGTCATTCTGTTACAGCGCTATCCATTACTTTCACCATCAGACTTATCACAAATATTG
TCCAATAAATCGGAAAATTGCGACTATGACCCCCCTTATGCTGGAGATTTGGCTAGTAGTTGCCAGTTGATAACAGC
AGTTCCTCCAGAAGATCTGGGGAAGCGCTTCTTTACATCAGGACTTCTGCAAAATAGATTTGTCAGCTCTACCCTGT
TAGATGTTATTTATGAAAACAATGAATCCACCATCGAACTAAATAATAGGTTGGTATTCCATCTGGGTGAACAACTT
GAACAACTTTTTAACCCAGTCACAGAATACTCACCGGAACAGACAGAATATGGTTATAAGGCGCCAGAGGACGAATT
ACCCACAGAATCGGATGATGATCTTGTCAAGGCCATTTGCAACGAGTTATTACAACTACAAACAAATTTTACTTTCA
ATTTGGTAGAATTTTTGCAAAAATTCCTGATCGCCTTGAGAGTCAGAGTACTCAATGAAGAAATTAATGGGTTATCC
ACAACCAAATTAAATCGACTCTTCCCACCTACAATAGATGAAGTCACAAGAATCAATTGTATTTTTCTAGACTCGCT
AAAGACAGCAATCCCTTACGGTTCCCTCGAAGTACTGAAGGCATGCAGCATTACTATTCCTTATTTCTACAAAGCAT
ATACAAGACACGAGGCGGCCACAAAGAACTTCAGCAAAGATATTAAATTGTTTATTAGGCATTTCAGCAATGTAATT
CCAGAAAGAGAGGTCTACACGGAAATGAAAATCGAGAGTATAATTAAGGGACCTCAGGAAAAACTACTGAAGCTAAA
GTTAATTATAGAGAGATTGTGGAAGTCGAAAAAATGGAGACCGAAAAATCAAGAAATGGCAAAAAAATGCTACAACA
ATATCATTGATGTCATTGATTCGTTTGGAAAATTAGATTCCCCACTTCATTCTTATAGTACCAGAGTATTTACTCCA
TCGGGAAAAATCCTTACAGAATTAGCCAAATGCTGGCCCGTAGAACTGCAATACAAATGGCTGAAGAGAAGGGTAGT
CGGTGTGTATGATGTAGTGGATTTGAATGATGAAAATAAGAGAAATTTATTAGTCATATTCAGTGATTATGTGGTTT
TCATCAATATACTGGAGGCAGAAAGTTACTACACTTCAGATGGATCAAACAGGCCCTTAATCTCAGATATTTTAATG
AACTCATTGATCAACGAAGTTCCGTTGCCCTCCAAGATCCCTAAGTTGAAAGTGGAGCGTCATTGCTATATAGATGA
GGTTCTAGTTTCTATATTAGACAAAAGCACTCTACGTTTTGATCGATTGAAGGGAAAAGATTCTTTCTCAATGGTAT
GTAAATTATCCTCTGCATTTATCTCTTCTTCGTCAGTTGCTGACTTGATTACGAAGGCTAGAATTTTGGAAAAAGAC
ACTGCATTTCATTTATTTAAAGCTAGTAGAAGCCATTTTACATTATATTCTACTGCTCACGAGCTTTGCGCTTATGA
TTCCGAAAAAATAAAATCAAAATTTGCCTTATTCCTGAACATACCACCATCCAAGGAGATATTGGAGGTCAACAACC
TTCATTTGGCTTTTTTTGCAAGATTTTGCAGTAACGATGGTAGAGATAACATCGTAATCTTAGACGTCTTAACCAAA
CATGACGATAAACATATAGAAGTTACATCCGATAACATTGTTTTCACCATAATTAATCAATTGGCCATTGAAATACC
GATATGCTTTTCTTCCTTAAACTCATCGATGGCCAAAGATTTACTCTGTGTAAATGAGAATTTGATAAAAAACTTAG
AACATCAATTGGAAGAGGTCAAGCACCCTTCAACAGACGAACATAGGGCTGTTAATAGCAAACTTTCCGGTGCATCC
GATTTCGATGCTACTCACGAGAAGAAAAGATCATACGGTACCATAACAACATTTAGAAGCTATACAAGCGACTTGAA
GGACAGTCCATCAGGCGATAATAGTAATGTCACCAAGGAAACTAAGGAAATTTTACCAGTGAAACCTACGAAAAAGT
CTTCAAAAAAACCAAGAGAAATTCAAAAGAAGACCAAGACAAACGCCTCTAAAGCAGAGCACATAGAAAAGAAGAAG
CCTAACAAAGGCAAAGGGTTTTTTGGCGTGTTAAAAAATGTTTTTGGAAGTAAAAGCAAGAGCAAGCCTTCACCAGT
TCAAAGAGTGCCTAAAAAAATATCGCAGAGGCATCCTAAGTCTCCAGTGAAGAAGCCAATGACCTCAGAAAAGAAAT
CCTCCCCTAAAAGGGCAGTCGTTTCATCTCCCAAAATTAAAAAGAAAAGTACTTCTTTTTCCACAAAAGAATCACAA
ACTGCTAAATCTTCTCTTCGAGCAGTTGAATTCAAATCTGATGACTTGATCGGAAAACCACCTGATGTTGGAAATGG
CGCACATCCTCAAGAAAATACCAGAATATCTTCAGTAGTAAGGGATACAAAATATGTCTCCTACAATCCCTCTCAGC
CTGTGACAGAAAATACCAGTAACGAAAAAAATGTCGAACCAAAAGCGGATCAATCCACAAAGCAGGATAACATTTCC
AATTTTGCAGATGTAGAGGTATCTGCGTCTTCTTATCCTGAAAAACTTGATGCAGAAACAGATGATCAAATAATTGG
GAAGGCGACGAATTCGTCATCAGTTCATGGAAATAAAGAGCTGCCAGACCTTGCTGAGGTGACTACAGCAAATAGGG
TTTCTACAACATCGGCTGGGGACCAACGTATTGATACCCAAAGCGAATTTTTACGTGCAGCTGATGTTGAAAACTTA
AGTGATGACGATGAACACAGACAGAATGAAAGTAGAGTTTTTAACGATGACCTCTTTGGTGATTTTATTCCTAAGCA
TTACCGTAATAAACAGGAGAACATTAACAGCTCGAGTAATTTGTTTCCAGAGGGAAAGGTGCCCCAAGAAAAGGGCG
TATCAAATGAAAACACTAACATATCTCTCAAAACTAATGAAGATGCATCTACATTGACGCAGAAACTCTCTCCACAA
GCGAGTAAAGTGCTGACAGAAAATTCTAATGAATTAAAAGATACCAACAATGAAGGGAAGGACGCAAAGGACATAAA
ATTAGGAGATGATTACAGTGATAAAGAAACAGCGAAAGAAATAACTAAACCAAAAAATTTGTTGAAGGAATAACTG
AACGGAAAGAAATATTCCCCACTATTCCTAGGTTAGCGCCGCCAGCTTCAAAAATTAACTTTCAAAGGTCACCATCC
TATATTGAGCTCTTTCAAGGAATGAGGGTGGTTTAGATAAGCATGATGCCCATTATAACTGGAAACGCTTGGCTAG
TCAAGTCTCCTTAAGTGAGGGACTAAAAGTCAATACTGAGGAAGATGCGGCAATTATAAATAAAAGTCAGGATGATG

CCAAGGCGGAAAGAATGACTCAAATTTCTGAAGTGATTGAGTATGAAATGCAGCAACCTATCCCAACTTATTTGCCT
AAGGCGCATCTAGATGACTCGGGTATTGAAAAAAGTGATGACAAATTCTTCGAAATTGAAGAAGAACTTAAGGAAGA
ATTGAAGGGCAGCAAAACCGGTAATGAAGATGTCGGTAATAATAATCCATCCAATTCTATTCCAAAAATCGAGAAGC
CCCCAGCATTCAAAGTTATTAGAACATCGCCTGTGAGAATTATCGGAAGGACTTTTGAAGACACTAGAAAATATGAA
AATGGCTCTCCATCTGATATTTCGTTCACTTATGATACTCACAACAATGATGAACCTGACAAAAGGCTGATGGAATT
AAAATTTCCATCCCAAGATGAAATTCCGGATGACAGATTCTATACTCCAGCAGAGGAACCCACTGCTGAATTTCCGG
TGGAAGAACTTCCAAATACTCCGCGAAGTATTAACGTTACAACTTCAAATAACAAGAGCACAGACGATAAGTTGAGT
AGCGGTAATATTGATCAAAAACCTACCGAACTGTTAGATGATTTAGAATTCAGTTCATTTAATATAGCATTTGGAAA
TACCTCCATGAGTACTGACAATATGAAAATATCATCCGACTTAAGTTCGAATAAAACCGTGTTAGGAAATGCTCAGA
AAGTTCAAGAGTCTCCTAGTGGACCATTAATCTATGTTTTGCCTCAGAGTAGCACAAAGCATGAGAAAGAGGGGTTC
CTTCGAAAGAAACAAAAAGACGAGCCCATTTGGGTTTCCCCTAGCAAAATTGACTTTGCTGATCTAAGTAGGAGAAC
TAAAGCATTGACGCCAGAGCGTAATACTGTTCCTTTGAAAAACAACGACAGTAGAAAATACAAATATACTGGAGAGG
GATCTATCGGTAATATGACAAATATGCTGTTAACTAAAGATGCTTCGTACGCGTACTTAAAAGATTTTGTTGCGTTG
AGTGACGATGAAGATGAAGATGGGAAACAGAACTGCGCTGTTGGTGGCCCAGAGAAACTGAAATTTTATTGAGGCGC
GCCACTTCTAAATAAGCGAATTTCTTATGATTTATGATTTTTATTATTAAATAAGTTATAAAAAAAAATAAGTGTAT
ACAAATTTTAAAGTGACTCTTAGGTTTTAAAACGAAAATTCTTATTCTTGAGTAACTCTTTCCTGTAGGTCAGGTTG
CTTTCTCAGGTATAGTATGAGGTCGCTCTTATTGACCACACCTCTACCGGCAGATCCGCTAGGGATAACAGGGTAAT
AT

Supplemental Figure S2. Fungal proteins similar to yeast Bud3 used for domain alignment. The full-length Bud3 protein sequence from *S. cerevisiae* was used as a query to search for similar proteins within the fungal kingdom using BLAST (NCBI). The following proteins were used to perform a CLUSTAL-W alignment:

NP_009914.2 Bud3p [*Saccharomyces cerevisiae* S288C]

```
MEKDLSSLYSEKKDKENDETLFNIKLSKSVVETTPLNGHSLFDDDKSLSDWTDNVFTQSVFYHGSDDLIWGKFFVCV
YKSPNSNKLNAIIFDKLGTSCFESVDISSNSQYYPAIENLSPSDQESNVKKCIAVILLQRYPLLSPSDLSQILSNKS
ENCDYDPPYAGDLASSCQLITAVPPEDLGKRFFTSGLLQNRFVSSTLLDVIYENNESTIELNNRLVFHLGEQLEQLF
NPVTEYSPEQTEYGYKAPEDELPTESDDDLVKAICNELLQLQTNFTFNLVEFLQKFLIALRVRVLNEEINGLSTTKL
NRLFPPTIDEVTRINCIFLDSLKTAIPYGSLEVLKACSITIPYFYKAYTRHEAATKNFSKDIKLFIRHFSNVIPERE
VYTEMKIESIIKGPQEKLLKLKLIIERLWKSKKWRPKNQEMAKKCYNNIIDVIDSFGKLDSPLHSYSTRVFTPSGKI
LTELAKCWPVELQYKWLKRRVVGVYDVVDLNDENKRNLLVIFSDYVVFINILEAESYYTSDGSNRPLISDILMNSLI
NEVPLPSKIPKLKVERHCYIDEVLVSILDKSTLRFDRLKGKDSFSMVCKLSSAFISSSSVADLITKARILEKDTAFH
LFKASRSHFTLYSTAHELCAYDSEKIKSKFALFLNIPPSKEILEVNNLHLAFFARFCSNDGRDNIVILDVLTKHDDK
HIEVTSDNIVFTIINQLAIEIPICFSSLNSSMAKDLLCVNENLIKNLEHQLEEVKHPSTDEHRAVNSKLSGASDFDA
THEKKRSYGTITTFRSYTSDLKDSPSGDNSNVTKETKEILPVKPTKKSSKKPREIQKKTKTNASKAEHIEKKKPNKG
KGFFGVLKNVFGSKSKSKPSPVQRVPKKISQRHPKSPVKKPMTSEKKSSPKRAVVSSPKIKKKSTSFSTKESQTAKS
SLRAVEFKSDDLIGKPPDVGNGAHPQENTRISSVVRDTKYVSYNPSQPVTENTSNEKNVEPKADQSTKQDNISNFAD
VEVSASSYPEKLDAETDDQIIGKATNSSSVHGNKELPDLAEVTTANRVSTTSAGDQRIDTQSEFLRAADVENLSDDD
EHRQNESRVFNDDLFGDFIPKHYRNKQENINSSSNLFPEGKVPQEKGVSNENTNISLKTNEDASTLTQKLSPQASKV
LTENSNELKDTNNEGKDAKDIKLGDDYSDKETAKEITKPKNFVEGITERKEIFPTIPRLAPPASKINFQRSPSYIEL
FQGMRVVLDKHDAHYNWKRLASQVSLSEGLKVNTEEDAAIINKSQDDAKAERMTQISEVIEYEMQQPIPTYLPKAHL
DDSGIEKSDDKFFEIEEELKEELKGSKTGNEDVGNNNPSNSIPKIEKPPAFKVIRTSPVRIIGRTFEDTRKYENGSP
SDISFTYDTHNNDEPDKRLMELKFPSQDEIPDDRFYTPAEEPTAEFPVEELPNTPRSINVTTSNNKSTDDKLSSGNI
DQKPTELLDDLEFSSFNIAFGNTSMSTDNMKISSDLSSNKTVLGNAQKVQESPSGPLIYVLPQSSTKHEKEGFLRKK
QKDEPIWVSPSKIDFADLSRRTKALTPERNTVPLKNNDSRKYKYTGEGSIGNMTNMLLTKDASYAYLKDFVALSDDE
DEDGKQNCAVGGPEKLKFY
```

QNG12518.1 uncharacterized protein GWK60_B04697 [*Candida glabrata*]

```
MVSEQSSNYSKESIEEKPILLHNNLAHTSPDVQVSLTEIKDTLTIFRGSDELIWGDFLVAIGMDNNTNFNSIMVNKF
GATTFNNVNISKNSKYYPAIENLDPKYKDSNARKCLAVTLLKIYPLFEKHITTVSDLPFEYDQTHAGELASTATLIP
NVDPEVFLLKLKNYGMLNDTSRVITSTMVDVVYENNEDVIDYNNQLVYYLGEQLEQLFNPVTEYSPEQTEYAYKAPD
EEPSLFNSDTSLLKSVCNELLQLQTKFTYDLVEFLQEFLIIILRVNVLNEEIEGLSTLKLNRLFPPTIDEVTRINCIF
LDSLKAAVPFGSFEVLKACNITIPYFYKAYTRHEAATKNFSKDIKLFLENFGDVIPKKEEYTEMKMEALMKGPQEKL
LKIKLIIERLWESTPDWGNQEKEATSFYNNIIDIIDSFGKLESPLHSYTTRVFTPSGKILTELAKGWPIELQYKWLK
RRVVGVYDIIDSSDISKRKLLVIFSDYVVFLDIQNSESYYKNPNRPMLSDILTNSLINEVPLPSKIPRMKVQKYSYI
DDVQVTIVDGNILRFDCIREVDPFSISCRLKSKSTTEKRIADLITKAKILEKDTAFHLFKAEVNGVTLYSTAHEYQA
YRSERQKSKVALFLNLSPSPSYITENSLYAGLFMKFEDTTRLDKIMITTVLYDGTKSNYVVRPEQMITFVVRQLSQL
LPNCYSSTRSPLATSLLNLQAQLISELVKPKIAASSKSNDSAKLMDMNNFIAEKADKSDKYDAKHEKKRSYGTITTF
RSYKSDLKDVESSGEIYSREHNTSTKISKNNVQKISKNVRTKVTESPGRKQRVSKTQADKSKKTSGLSNFFKSIFKG
SGKKTKRPTNEKIQLKRIGSNKNISHVHISSKPRNKAVIKDTTNTAELEEPLISRAVTSVEISAEKVDRKDEDERVL
SVVHNKQFEETKANSEAPADNERMGQDIHHDAGNTSDLLIKEIADEVVEQKEFLEQLSKNMSIQKENKTVQSQVTQK
PTSIKNQASALYLYDDDLFGDFKQKPNDHDNISVSLENELTAEGSENEDEDPKKVSHQYQEETIEYQENEPKNDEPD
NRSKEADEQADSKENVELLPTKSEVSPGKPLVFPTIQKVVKKPQQIQRSDSFYELYKGMRMVLDDTDVKYNWKRLPS
LVSLSVQNAVNSDKSKHAFEKIAHARETPLLVENLMEKKELPMQATETQVREARKLKDAAVIKPVENNFKPVSDTLK
SPFKAYPELTKSTEYVNNDSINIFSELEKAFEVPQAKVSVPTFKVVRTSPTRYVQLESGSIRTDRMVPSPELSQSLS
YSSFVEELQNKKPQRLVEIADSITDMQSNLTKDDETIASSNAPSHVSSEFEQQKDISQQSDSSLEEEHYSAKKPLSQ
EINSSGILESLEFSSFTMGFEDTIDVVNSSHIPSGDLLPETGSRNMVLTRPKRNDAHPVYLLPRYSISTTKSLNQTR
ERMQYDEDAIWVSPTKLSFSEKDSSIRLVKEGTTPTKTLTHQGNKTKVDIPREESSFGYLSSLLAVDSNNNNILEFV
```

XP_003959137.1 hypothetical protein KAFR_0I02230 [*Kazachstania africana* CBS 2517]

MNDLSSIYSQELELTERSVVISLFNNLLASKNTHQQTLDWIHERSEKEWLDDIFINSVLYHNVDRVIWGPFFICIYK
DPKTDKFGSLTLDRFGITHINSIDLSRKSAYYPAIENLHDNDKNSNVKKCIAVSLLQKFSNISMQHLKYLTEDKINY
DPVHAGDLSSGCKLVTSISPELFGKRLISAGLLTGRLINSTLMDVIYENNESTIDSNNRLVFHLGEQLEQLFDPVTE
YSPEQTEYGYKPPEDDKPTETDSELVQAICNELLQLQSNFTFTLVEFLQKVLIALRVKVLNGEIDALSTVKLNRMFP
PTIDEVTRINCIFLDSLKSSMPYGSLEVLKACSITIPYFYKAYTRHEAATKLFSRDIKQFLKHFRNSIPECEEYSEM
KLEAIIKGPQEKLLKLKLIIDRLYHSKEWANEENKIIGKKNYDNIIDVIDSFGRLNEPVSSYSTRVFTPSGKILTEL
AKGWPVELQYKWLKRRVVGVYDIIDQTFSNKRALLVIFSDYIVFLSITDYELYYTDDGSNKPLISDILMNSLINEVA
LPSKIPKLNVQNYCYIGDVLVSIFDNDTIRFDALRPDAFSISCKLATMKEPIDAKKVAALVTKAKILEKDTAFHLFK
ACRDDISLYSTAHELEAYNNEKLKSKFALFLNIKPASQFLSLYNLHFAVFASFVGTDDTNKVRLSVITRSNKDTVKT
LEIFPDNIVDSIIGQLSTEYPLCYSSIQSSLIKELFAVTTYLANSIGKVGEKEHTLREAAAIVKDSNKSSKTNTLVN
SDAEKKHSKAKDIDAKASKKIKKKTDNSTQKRISKIPESKNVMNLKPQEVKKKSLIEKLKSIFKSKRRSKKDISGPI
VVNSKSYSSKSSLPHKKNNSPLPISPNKNILANTRSEKNLNAKNSSGKNPGVKSNDDAENLRISSVVRDTTYDASGA
RFQYTPRIGDLSKEKTEDIAEPHTPDEYTQLSQIPSPHIAEKLFLPKESKIHEPVDEVSEHEDIAGDISALTQSTSQ
ENVEVLSAGQSGKEKDVSKRAHNQSQLYNSDLFEDFVPPGKECGHNIEIKQASEELSDDNLPTEKSSTQNQLHNVEK
NNAVTLNSGEDNTKEKKNGTFHAGKQDPFVDIANPPIVQENIGVLPKKLNIFPTIPKLAPISRMEFTRSPSLIELFE
GMRVVLDETDAHYNWKRLSSEVSLNQKYITNSGNTPENNNNFRNFAHAAAFNLAIPKNVETIVIPDEKAELESSHST
NGTLKTGDDHSKTTVPSVEEVSEEAVGKSPLKHEENPSLAANDTNRDRALDSERKFKVINTSPTRYSNIGSSEKQLA
STTSETDFSKASFENPEDLQLSIARDISSQKLSVDTTTKMVPDFSFLTEMNREVTTRLFELNLASQEDVNDEEYYTP
TTNAPKENMAASTSDQVETENETSIGLDVATGLENDLAKIRETSETHPEGDKSQNVATEESQNFLEDLEFSSFAMTF
DNSLFNQYSSGLQEATLTHNSYETTKILDNIPELPAEKEGPVVYTLTNDLFSSNDIRIGINQKAGFMDNSNDAEDPI
WVSPSKLDFDDITRTHVRTLGENIKDHAITRKNTKPDPPSNKLDDSELKHDLSYAFLANLVQTSEFDEIEEENYNDD
KPTRLQFKS

KAG0668095.1 hypothetical protein C6P45_004993 [*Kazachstania exigua*]

MVMADIEINEFSSIYSHDSERNKHIDHLVQTTDIQGSSTPEINQSEIPKNENDIVLFNNYLSGADDSTTYNDIQSFS
KIKSYWTQDVFDNSKIYTSYDDLIWGKFVLCIHHSKDTYNCLMMDDFGIRELLNIRTSLESLYYGAVEGLNSDDKNS
EIKKCIAILLLRRYSELSSIQKIELKSLYPTRSIEQYDPAHAGQVASGCIEVKEVTASKFGERLISQGFLDKHIVKS
TLIDVIYESKESTIELNNRLVFHLGEQLEQLFNPITEYSPEQTEYGYKAPDNDIATETDTQLVEAICNELLEVQSNF
TFNLVEFLQKFLITLRVQVLNNEIEGLSTVKLNRLFPPTIDEVTRINCIFLDSLKSATPFGSFEVLKACNLTIPYFY
KAYTRHEAATKNFSKDIKLFIRNFSDIMPNKDIYTEMKLETIIKGPQEKMLKLKLIIDRLYKKKVWSAENSNNAKKY
YDNIIDIINSFGHLEAPMSSYNTRVFTPSGKILTELAKGWPVELQYKWLKRRIVGVFDIIDSNNPSKRNLLVIFSDY
IVFLNILNYKKYYTSDKSNKKPLISDILMNSLINELPLPPKIPKLEVEKYCYINDIHTSVTNGDILRFDAIRGKGKQ
PFSMVCQLASKTNSADDIADLITKAKILEKDTAFHLFKSVIKESTLYFTAHELESYNTERIKSKFGLFLNMEPSNEL
LISNDLHSAAFAKFTDPEKSNMVKLTVIRRHDRLSSKTDTIPADDIVCTLMQQLSSDIPICYSSILSEDAMKLVCVN
NIVTNKVIEEELFENITDDISTQNIASQVNEENKKSYGTITTFRSDVSDLVDVPHMKEDKRRDADGTSANKASETKK
ESTKTKAPKSAKKSSRTDKTNQKKTRATNKKTDDKKSTPKERKNKGFFGAVKSIFSNSSRKEKRTIGKPVIVKQKTT
NRDLEVKQHIKSKSHPLSPVKVTKPSDSKTSDEKSKLEEVKVEKNDRKVDKTEKDDNSEEDDKVEERKCSEIYNVRI
SSVVHNIEYTGSSVNTQQGSNKTEITNSIPKVAPAFKIMSPTKELPDIPVKVENDDLVISDVSRRTDSFDETEGNDT
DIKLANPITHQSKLFDNDLFGDFIPEKVHSKQQEIEKASPEKKDKIEVHPESSEFKLTLNTPVDIPAEHNIELPTLD
DINLEPNDDINEGLLTDIHEPRIKKKVQIFPEIANVEPPRSRIKFEKSPSFIELFRDMRIVLDDSDAKYNWKRLSTE
VSLNEKYLVNNEVPSSENVSNKHGLKTIVESNYNLPQKQSPMMRLSNVDPVSPARQKESHSETSPFRALAHNKLSDL
TVNDLHKFNSEAKNENITLRTPLKNPVNNASPNIGSPSARSSPLKTGSIFKVINKSPTRITNSPSKEATMELSHNLK
ENRKFLEQQALSTGMNLQPPPLMNSKSNNMNERNIASDFSFASDFNGDSNRRWVKLNVNSKEDLLDDTFHTPLEEPS
ETFSDALFGDINVTTNSAVPQLPQIPAKQTNSQIAAVVDINKNSSQGNHSKEKADILDDLEFSSFDMTFNTSTTTND
MEPTVPTDMSNSKNILVGLNNNDIPKMDPPVVYKYSKEQSFNTRKNLYNDTKYDDDSNEPFWISPSKVDFTSLTKSI
TKPTRNGTTLNPNIYTADAHTETPMKRNFKSTNREINLTQDMSFAFLGSLVDIDGTGDNLDDQAQKMYLHVYNLSNR
DIYKN

SCW02591.1 LAFE_0F09868g1_1 [*Lachancea fermentati*]

MENGAKEGLIDSSNATVDKMDTGCRIKLRPNLALNGKPKQWIIDRSTEEWVSQIFPTAAIFKGYDNLIWGNFFIVVY
REILTEKFSSIIIDKLGLTHFNSIDISRQSRFYPAVENLTDVNQKSNVRKLIAISLLRKYADIDSKLIKKISPEMKY
DYDPTIAGDLANSCELFGQCTPEEFGESLSQLGVLRDRFINSILLDVVYENKAKIIDSNNELVFHLGEQLEQLFNPL

SEYSPEQTEYVYKSPDDGYVVESEDPLIVSICNELLQLQTNFTLSLVEFLQNFLIPLRIEVANDEIDGLSIPKLNRL
FPPTIDEVTRINCIFLDALKSATPYGSAEVLKACSVTIPYFYKAYTRHEAATKNFSKDIKLFLSKFGHLTPNRDVYT
ELKIETLIKGPQEKIMKLKLIIERLWSNENLVHDDAAKKRYDDIIETIHSFGTLEKPMSAYSTRVFTPSGKILTELA
KGWPVELQYKWLKRRVVGVFDMVDADDKAKRDILVIFSDYVVFLRVIGGEDYYNMDGNKPLISDILMNSLINEVPLP
SKIPKLEVLAHTYIDKVLVSTYGSSFLRFDLRDKNFCTPLTYELASNSMTTSEVADLVTKAQILEKDTAFHLFKYSN
DYLQIYSTAHELSAYSTEKIKSQFALFLNLEPSIELIEKYGLCMALFASFRKDHQVNLTRITVDNARTELTVPLHDL
AETLANELVDAIPIYFSSLSSPFYEELLQLNAKLVQRIGKSFRKDELTIQENKNDKAPEKTSSAEIIYHHQKNKSFG
TITTFRSFPSDMKELSDNNTINEKIKDKSKHGGSKARKTVNRDIANHPPKQMGLIKTFKNLFGRRKKSRKENERLTI
SSPHITSPKTESKANSSITRVKKTIGDPKSHVRESQEVEHLATQPAESSRVSSVIHTPSRNITDCVDISKESMSSMN
RKAQAGVEKNSTENSSSTQLQDYEINHSSLSVHSEKEVLPDSVKATGTPYKESLRQSKVFNDDPFASVISSDENESI
GPAKDNLCGESLTGETSVSVKDSKLVGKEADEPTIHKEDNHFAKTLAEKVELKPSIPEVHDTAKSFEGPIDSRRLSE
GRCGQEFENAQIFPKVQGLKTKTIDFCRSPSFVELFDGMRAILDITDESTNWRRLSSEGSLTIQTTPHAEDVKALDK
NCIKGNSSAIMLDKSNHSNMPDARKSSPDHRDENDDARIFASSKVSEPEYGQLNLDFSKSEGTLTPVFPTFKVVNTS
PAKIVNFATSINNVHPDLTNNSTRESSSPVKNSYSSDLNAENLRLVELSFNSQDDVYSYDSRQKPNDILETLPPSHS
IHSPRLSNSEVNSKPLADQLSATTSVEVANLQNELVIEPLESNILGDLDFSSFNMTFDISNELNDSTQESIMDEPKN
PFVRKPHLSPRDPVFYRLPNSTRSDETFFSCVDDQRNKGQKRKSYINSLPLECEDEPMWVSPSKIDMFDLSKQPDSV
FQELKLRSKQTREAKFQLPGKANRSSTEEQLLLPDSSYAYLGSLLTDDDMQLAEISSDDRPVRLKFNP

XP_003980081.1 hypothetical protein NDAI_0G04200 [*Naumovozyma dairenensis* CBS 421]

MATTQDLSSLYSNHNNDITGKENANTLTIPLYRNLIESYLPESISIGNHFLQNEKQLNACFDDSSHEASRYAGIFKG
FDELLWGQFIVCLYKETASKKYNAIMLDKLGPTTFNSIDVSKDSQFYAAIENLNTTNRKSTLRKAIAVILLQKFVEI
PKEGVHKIIQPNKTLNFEYDPQYVGELTNSCEEISSSNHNNSSNSYQVISFMKKLYTMGSFQRKYINSTLLDVVYEN
NESTIEINNRLVFHLGEQLEQLFNPVIEYSPEHTGYVYKPPDEEESTSNISNNGNDNSNGKNDQEDNALIKAICNEL
LELQTNFTLSLVEFLQNFLIVLRVKVLNDEIDRLSTRKLNRLFPPTIDEVTRINCIFLDSLKAAIPYGSLEVLKACS
MTIPYFYKAYTRHEAATKNFSKDIKSFLRNFDDAIPKNQSLTEMKISTIINGPQEKLLKVKLIIDRLYTSKKNWNIP
NKTKADKYYNNIVDVVDSFGKLSNTSLSSSYNTRVFTPSGKILTELAKGWPVELQYNWLKRRVVGVFDIVDETYGGR
KKLLVVFSDYIIFLNIINPDSYYSIETDNQDNSLIKPHLSDILMNSLINEVPLPAKIPKLEVDNYCYIDDIFASTFE
ENYLRFDAFKEDKSFSVSYRLSTESTPVSTVADLIMKAKILEKDTAFHLFKASFNNITLYSTAHELAAYNTEKIKAK
YALFLNIEPSKELLIQNDLHMAIFAKFANENEGKFINLDILTSTEVHRKDTFEAAKIIPELIGQLLLEIPIYRSSIV
SSQAIKLMAINEGLVKQCISCLEKETPSIPITDSYNKPAQTDKQAKEKPYETTLTSKNIVNNSRALRGKSVNTKTAV
EKDNTVKKIEKNTNKKLPSAKNLPRKQTGKLPTNVPSQLLAKKEKRRSGLITAFRGIFGSKKNKDAEKGRNQLDDKS
KNYQKTTPRQKLVPKVSQKDSIRKENTTPQPIKNESVTSFARVSKNQKIEPPMLSEEQRITSVIHNRQYTEDIQGNK
VMNNLDSESKENVLSETATTTPLTSKEISVSSDKQLRTALESPPGSIGNSEKVLPDPNRSLSNKNTTSRMPQIQIFT
DDLFGDFVKPPSLDKQTAKQEETECDNGDEDAEHYQKHLQEDALGEGVSSKDNSPLPTTANATSENRGFIAPSENPS
FRDGEIQHEKVKIFPDIPKMQLSKIQFDRSTSFVELFKGMRLVLDDSDAKYNWKRIPSEQGLNINNLVNNATTIKNT
DRNMVGNEIEKVIPKEKCYSEAAQEQRPSTPESSSNITEAEEEPREEKEGGHITSPQPVLSTIALLPDQTRRRLPTF
KVTKTSPVRIINKTLNSPQKVVGKVENDSCFSSDVTIGNIENNKRLHKLTFRSQEDVFDAKICTASRENIDTIEATV
QADRSKNNEEQSSKLHDRLARSGSNENLLEAFEFSSFNMSFQDLPENNEECVTSIHPVTIHDTVVPPLQNLEPAPMV
YRLSNEHSSNKNKLEADDDPIWVSPSKLDLLSSNKLRDRVLVNKTENEALSNQKTPIASIKNKSIPSQKLLDTSLIR
NASYVYLTDFVDFHDDEKYEDKPTRLQFQ

XP_003679944.1 hypothetical protein TDEL_0B06040 [*Torulaspora delbrueckii*]

MTEVDLSSLCSATPKDAVKLHGESMKVRLFENLLERHIKNRQWLKDRTGEEWVEKVFKEAAFYEGVDELIWGQFFIC
VYKDPQSTKLSALIIDKFGLSHFNPVDISPKSQFYPAIENLDPKERKSNVRKCIAVSLLQKYAQLPDGCIHRIQPNG
LKFDYDPTHAGDLASACKLVDFCTPENAGKSIKSLGILGRRHVQSSLLDVVYESNSNNSEENNRMVFNLGEQLEQLF
DPLSEYSPEQTEYIYKAPENDESLYQDNQLVKSILNELLELQMAFTLSLVEFLQGFLISLRVKVLNNEIEGLSTVKL
NRLFPPTIDEVTRINCIFLDSLKSATPYGASEVLKACSVTIPYFYKAYTRHEAATKNFSKDIKLFLRRFQDLIPEKD
VYTEMKIETIIKGPQEKLLKIKLIIDRLYEEKEWPEELQEEAQRNYNSVVEVIDSFGKLEVPLSSYNTRVFTPSGKI
LTELAKGWPVELQYKWLKRRIVGIFDVVDANDSSKRKLLVIFSDYVVFLDIARAELYYAADGKNRPQISDILMNSLI
NEVPLPPKIPKLTVNSYCFIDDVLVSLVEGNSIRIDALREGESFSTTFRLASNSTTASTVAELVVKAKILEKETAFH
LFRAMQDNMTIYSTAHELEAYQNEKIKSRFSVFLNMKPSKDILHKNRLHLAIFLKFVTVGQNEQIQLEALTSDERTR
KATFPPEELVPALIEQLSIEIPVCYSSIYSPLYSILIEINDLLVKKIGHHFNPPIEEKDLANSDQASSFIREHEKKK
SFGTITTYRSHVSDFKDATNEQTNSGESHSTHKIPARTTDKTTKAAANHQKLHSSRKIDNAKKRRSIVGLVKGIFSG
KRSKDDVIKNKSKSTTKQSPKMRSKNMRADALVISKPTISKPNPPKNETEIYEKSQRISSVIRKTEFSPVEDPLINA
IEEPKIGSGATTKPALQNEAVSQNPLDVDISIHSSTIEPNLVERVEREEASSNHSTIRIAESAKQFYRQAGRQSQLF

NDDLFGELGSEPATEANQKQVDSAKIESKPKRKEMITRQDSSTLDSHTEGSGGSDFDEAPHTKNTDEGEMSTAHCFD
TETGRDSSAKTQQEVEKLVQDDIVNQKKIQIFPSISPPKLSRINIQRSSSFIELFEGMRLILDESDAQYNWKSLSND
GFLREVQESDEPATIPHVFRPIAIANTKLIPDETENLLNGKPSLSLNQCEDMFENQESQEISSPALKTNNVSPDASK
AVIKESSVDSRSSDPSKVKSPRKNGFKVVKSSPTRIIKKPFQQINIEVPDQNMTYNFSISSDLNQGADKRWFELKLP
SQEDLNSEIFHTPHEEPSSEFPEEQHNSETPSPFESDPNVIETSQDTITTDKPLQKGQDALLEDLEFSSFHMTFDTA
EGNSEQSPDTSSSDAAGATSSPKANSLVQNNKKDGPLLYRLPMTFSTKLSQSERAPTGDVGEDDDPIWVSPSKLDFY
DLSNTADSGATNYDLKVSARRDERKEPSIGDENMGTNRYSLRELSYAYLASLVSPTETSFEVDDKPQRLQFQ

XP_001646494.1 hypothetical protein Kpol_1048p67 [*Vanderwaltozyma polyspora* DSM 70294]

MRQVSYTSLYSDTVNVAAPFNNVNKYLDPTKGEIDAQQGSVSNIELFKNLIQYKLKDDKYQIEKTDKNWLDQVFEGS
KIYYGLDEILWGKFMVCVQCDKLNRKQTVLILDKLGVTFSDVIDVSNESQFYPAIENLDPKHNNSLERKCIAVALLQ
KYSLLSEEEISKIKSTVVKNNYDQIHAGDLASRCEILETKDVKKFGETILNTGLLQDRTVKSTLMDVIYKNESTSIE
LNNRLVYHLGEQLEQLFNPLTEYSPEQTEYTYKPPEEEKIDNTDSELTKAICNELLQFQTKFALNLVDFLQNFLIAL
RVEVLNEKINGLSTVKLNRLFPPTIDEVTRINCIFLDSLKAAIPFGAFEVLKACSVTIPYFYKAYTRHEAATKKFNK
DIKLFLANFGKQIPSPQVYTEMKIETIIKGPQEKLMKLKLIMDRLWESQKWSDEEKDKAKEYYDNIIEIIHSFGTLK
EPISSYSTRVFTPSGKILTELAKKWPVELQYKWLKRRVVGVFDVVLGPEFNDRGLLVIFSDYIVFLDIMDAHDYYND
KESNKPWLANVLMNSLINEVPLPSKIPTLNVSNYCYIDKVIVSVYERDIIRFDSFYDSDPFSISCKVISKTTSTNLV
ADLITKAKILEKDTAFHLFRANENGILLYSTAHEIDAYEKEKIKSNFSLFLNIDPSAELLDNYNIHVAFFAKLIDRP
NGETIQLDILTRDNRKYQVNISPDKLIPEILHQLSYEIPICYSSLRSNNFSDLLNINKVIISNLITGSVDKKDIVDE
PNGKTSADSFTMIHSKHRSYGSITTFRSYKSDMKDGSAEPIKEKVDATTPIENKRDTIRTQPQTILKDSGKTVPKTK
TVHSQKQAVKTEVSAAKEIERPRKSNVKDNKKKEKRKSFVGAFIGLFGSKEKKNKSSERNNNVKSKEGSKSFLKRKQ
KQKASKKTISKPQAIDKSGESNKIADNNLNIAERNNKSSERHEDKIEDSEQQRVSSVIRNKDYYSSKDNQPATVEKA
EMVNKRTDNKVIELKKSEILTKEVDGIQNNSKISTKDIFDKSFNDDLFGNYKVGNDTTVDKVKEQNNQDSNPNTENL
KQNTSSKHVPIQTTIPWAKDIDTLTFALNITKEDNNSKLVDTDETSKNEIHTEKAIEQTSKSDSKIGTIVDKDNEDA
ILVEKNIRMKDQSIFPVVPKLIQKNIDFSRSASYKELFEITRLVLDELDAQYNWKRLKQEASLSENHVISASFGSTE
DLVETEIESKKDIDNVTPEVVKSSDSNKILKISDIPILEPDSSLDNSEITKDFLNDLENIVDDVLKSNDFKKQSPSI
QNQHKVFKVIKTTSPTMVNKNFDKKSSTDSNSDARSNEIPKKLLPSVDLVPGIKLEDEEEEEDQYLTPSAEPSAVFE
QDMENETSKDSSDESTVNDTNKSVRAEILEDLNFSSFHMSFDDSKENEVENSVNLGANSELLNQLTKKNDTFQSPFA
GQAPLIYRLPKSSPIKNTANNISGAKKLNRDDNDAIWVSPSKIPFYDFNGKPESNVQGKKSKAAVQEQLTNLQISGN
SNMNKDISYTYLAGFIDSPDLGESNSNRLKFKE

XP_037143118.1 uncharacterized protein HG535_0B04320 [*Zygotorulaspora mrakii*]

MLERRENSLEMTAADLSSAYLHEVRHEMGHADPDAATDIILFGNLLEHEVERRQWVKDRTPKEWIQQVFRDSAVFHG
IDELIWGEFLVCIYKDPITNMISALFIDKLGVTHFNPVDITNKSQFYPAIENLTDNDKDSNVKKSIAVSLLRKYAQL
SSKDVSRLQPDGLRFNYDPTHAGDLASRCKLVESCAPETMGTRILSQGSLQRRCNKSILLDVIYERKESLTEINNKL
VFHLGEQLEQLFNPLTEYSPEQTEYTYKPPDYDPMSHRTSSKVVSIIDELLQLQTNFTFSLVEFLQGFLISLRIKVL
NEQIKDLSTVKLNRLFPPTIDEVTRINCIFLDSLKAATPFGALEILKACSATIPYFYKAYTRHEAATKNFVKNFKLF
LKSFEYCIPRDNGYSNMKIESIINGPQEKLVKLKLIIDRLYRTEEWPPNEQEEAKSNFDNIALAIDSFGKMKSPLSS
YNTRVFTPSGKILTELAKGWPEELQYKWLKRRVVGVFDVVGNDENKERQVLVIFSDYIVFLEVVNGSQYYSGTNNNK
PKIADILMNSLINEVPLPTRIPKLKVHNYCYIDDIIVTELENQSLRFDAYRGVNSFSVTCRLSTKSASASTVAGLVT
KAKILEKETAFHLFKANLEENEIYSTAHELEAYKNETLKSKFGLFLNMNPSRQVLAGSRSHVAIFASFISDMDASDV
QLTILTSDGRENASVVSPEDMILRIFEVLKHEVPICYSSLYSPLLPALLKINEQIVHKVLYSADPSDKFTNNTEGDA
LTFTQDHEKKKSFGTITTFRSYTSDYKDVESNQSRVSRSGTAKNETLGVEKSHKIEVKAPKAINSKNKGRINSKKRK
SIIDVFKGLFEIKRSGKKEADIAGTVKISKPIVKRGTNERLPKKKAASQEVKDPLKQREHKTGGDRITSVIKHKEFA
SNEGIDIASQNTSKNEPNEHIRNNILKEQKSFESISRDPAEKSSKDRSIATAQDEDSTAQGPSGVAQATSLRESVHS
ILSQTLKLPETPESRKVGDCAAPNGVQIHSAGQNGFYRGQSRQSQLFNDDLYGELAVEAAKEKSSDSSAETVSGHNT
KEVDAQKNHPDTTAPIQIIESPTIKQITPSEQIGSEKRISTVENFEVNISQYDGKETTEKNRIFPIIPTLDPPKLGF
ERSTSFVELFRGMRLILDENDAQYNWKRLSSESSLNEKQAFIAPIQNAHIFKTLAQDPDRRLGPPQEGNQIEMVKIP
SSPTGKLSTEDSSGIPDLANDTSARKLNNSNQSVVLQNIPGLPLDDSESKLNTQPKPSFLAAPNFKVLRTSPTRIVN
KLTKLHEVNRDSLNYDISLPLDLDMVSNKRWVELELPSQEDLQGDKFYTPAEQHNSEFPKEALEIDDHSTASEFAKE
ANETSQETSIEDSSKLIDAEDLLEDMEFSSFHMTFDASVSKDDISNYTSSPVQHDNNVNPQCPTTRHVRQEPVLYRL
PKDIFSTSRTISQLNDGGIGERIGHGRNPSLDDDPIWVSPSKIDFYNLSKDSEESTMANRKAAALLEYPYDNKTYEH
HNEDTNSLRELSYAYLASFVGSDQESERFDDKPVRLHFCD

REFERENCES

1. **Finnigan GC, Takagi J, Cho C, Thorner J.** Comprehensive genetic analysis of paralogous terminal septin subunits Shs1 and Cdc11 in *Saccharomyces cerevisiae*. *Genetics* 2015; 200(3): 821-841 DOI: 10.1534/genetics.115.176495
2. **Sikorski RS, Hieter P.** A system of shuttle vectors and yeast host strains designed for efficient manipulation of DNA in *Saccharomyces cerevisiae*. *Genetics* 1989; 122(1): 19-27 DOI: 10.1093/genetics/122.1.19
3. **Andersen MH, Graversen H, Fedosov SN, Petersen TE, Rasmussen JT.** Functional analyses of two cellular binding domains of bovine lactadherin. *Biochemistry* 2000; 39(20): 6200-6206 DOI: 10.1021/bi992221r
4. **Shao C, Novakovic VA, Head JF, Seaton BA, Gilbert GE.** Crystal structure of lactadherin C2 domain at 1.7A resolution with mutational and computational analyses of its membrane-binding motif. *J Biol Chem* 2008; 283(11): 7230-7241 DOI: 10.1074/jbc.M705195200
5. **Goldstein AL, McCusker JH.** Three new dominant drug resistance cassettes for gene disruption in *Saccharomyces cerevisiae*. *Yeast* 1999; 15(14): 1541-1553 DOI: 10.1002/(SICI)1097-0061(199910)15:14<1541::AID-YEA476>3.0.CO;2-K
